# Ending a bad start: Triggers and mechanisms of co-translational protein degradation

**DOI:** 10.3389/fmolb.2022.1089825

**Published:** 2023-01-04

**Authors:** Tom Joshua Eisenack, Débora Broch Trentini

**Affiliations:** ^1^ University of Cologne, Faculty of Medicine, University Hospital of Cologne, Center for Molecular Medicine Cologne (CMMC), Cologne, Germany; ^2^ Cologne Excellence Cluster on Cellular Stress Responses in Aging-Associated Diseases (CECAD), University of Cologne, Cologne, Germany

**Keywords:** translation, quality control, ubiquitin, ribosome stalling, protein folding, membrane insertion

## Abstract

Proteins are versatile molecular machines that control and execute virtually all cellular processes. They are synthesized in a multilayered process requiring transfer of information from DNA to RNA and finally into polypeptide, with many opportunities for error. In addition, nascent proteins must successfully navigate a complex folding-energy landscape, in which their functional native state represents one of many possible outcomes. Consequently, newly synthesized proteins are at increased risk of misfolding and toxic aggregation. To maintain proteostasis–the state of proteome balance–cells employ a plethora of molecular chaperones that guide proteins along a productive folding pathway and quality control factors that direct misfolded species for degradation. Achieving the correct balance between folding and degradation therefore represents a fundamental task for the proteostasis network. While many chaperones act co-translationally, protein quality control is generally considered to be a post-translational process, as the majority of proteins will only achieve their final native state once translation is completed. Nevertheless, it has been observed that proteins can be ubiquitinated during synthesis. The extent and the relevance of co-translational protein degradation, as well as the underlying molecular mechanisms, remain areas of open investigation. Recent studies made seminal advances in elucidating ribosome-associated quality control processes, and how their loss of function can lead to proteostasis failure and disease. Here, we discuss current understanding of the situations leading to the marking of nascent proteins for degradation before synthesis is completed, and the emerging quality controls pathways engaged in this task in eukaryotic cells. We also highlight the methods used to study co-translational quality control.

## 1 Introduction

A typical mammalian cell expresses more than 10,000 structurally and functionally diverse proteins, with copy numbers varying from a few molecules to millions (Beck et al., 2011). To reach their functional state, proteins must generally fold into appropriate three-dimensional structures, assemble with partners, localize to specific cellular compartments, and exist at appropriate concentrations. This is an enormous task for the cell: altogether, proteins dedicated to functions related to the life cycle of the proteome (translation, elongation, folding and proteolysis) constitute a remarkable 10% of proteome mass; with 3% dedicated to protein folding (Muller et al., 2020). Protein synthesis is inherently error prone. During folding, newly synthesized proteins must sample diverse intermediate conformations, at risk of assuming kinetically trapped non-native states. Moreover, stochastic errors occurring at the level of transcription, mRNA maturation, and translation can generate folding-compromised protein variants. The same is true for disease-associated genetic mutations and for DNA and RNA molecules damaged by environmental stress, such as UV irradiation. Certain proteins require translation at specific cellular locations, such as the endoplasmic reticulum (ER) and mitochondrial membranes, which involves the concerted action of specialized targeting factors. Mislocalization of nascent chains therefore represents another cause of defective protein synthesis. The protein folding problem is further exacerbated by the fact that many proteins require conformational flexibility to function, and therefore are only marginally stable under physiological conditions. Consequently, their structural integrity is challenged by proteotoxic stress situations, such as high temperatures and reactive oxygen species, as well as by a number of pathological states and aging.

If left unresolved, defective protein species pose a substantial problem to the cell. Misfolded protein forms are not only dysfunctional, but they also tend to engage in non-productive intermolecular interactions, forming potentially toxic protein aggregates. These aggregates, which in some instances are thermodynamically more stable than the native state, can overburden folding and degradation factors, thereby enforcing a self-propagating cycle that ultimately leads to proteostasis collapse and cell death (Hipp et al., 2014). Many pathological conditions are fundamentally rooted in the protein folding problem, including loss-of-function genetic disorders such as Cystic Fibrosis and aggregate-deposition diseases, as in many age-dependent neurodegenerative disorders such as Parkinson’s and Alzheimer’s diseases. For these reasons, cells largely benefit from early detection and rapid resolution of non-native protein species (Rodrigo-Brenni and Hegde, 2012). This can be accomplished by two general means, chaperone-assisted protein remodeling and protein degradation *via* the ubiquitin-proteasome and autophagy systems. The timing for transitioning from refolding to a degradation strategy is critical towards establishing an effective balance between overall protein synthesis efficiency and risk of aggregation, and poses one of the key problems in cell biology (Rodrigo-Brenni and Hegde, 2012; Duttler et al., 2013). Of note, the appropriate balance may vary between cell types and different physiologic and metabolic circumstances, adding another layer of complexity to this task.

## 2 How efficient is protein synthesis?

The overall efficiency of protein synthesis and maturation has been a matter of debate. Defective ribosomal products (DRiPs), defined as polypeptides that never attain native structure owing to errors in translation or post-translational processes necessary for proper protein folding and maturation (Yewdell et al., 1996), have been estimated to represent upwards of 30% of newly synthesized proteins (Schubert et al., 2000). In certain conditions, up to two-thirds of proteins were degraded during or rapidly after translation (Schubert et al., 2000). These estimations were based on pulse labelling experiments comparing the levels of newly synthesized proteins (i.e., those containing a pulsed radioactive amino acid) in the presence and absence of proteasome inhibitors over a period of up to 1 hour. However, subsequent studies raised concerns about this methodology. Proteasome activity promotes the recycling of proteins into free amino acids. Incorporation of a radiolabeled amino acid into newly synthesized proteins is most efficient in the absence of the corresponding unlabeled amino acid. Accordingly, reduced amino acid recycling from the unlabeled proteome during proteasome inhibition promotes higher incorporation of radioactive amino acid into nascent proteins, especially if experiments are performed in amino acid starvation media (Vabulas and Hartl, 2005). In the relevant timespan for DRiP degradation, it is therefore difficult to distinguish if eventual increases in pulse-labeled radioactive proteins in the presence of proteasome inhibitors stem from higher labelling efficiency or from blocking their degradation. Taking these limitations into account, Vabulas and Hartl estimated that only a few percent of proteins are degraded immediately upon translation, with around 20% degradation in the time frame of 30–60 min. Even in the presence of the proline analog l-azetidine-2-carboxylic acid (AZC), which promotes misfolding, proteins were protected from very early degradation (up to 12 min), but steadily declined at later stages (∼40% degradation between 15–60 min). The authors propose that proteins that are unable to fold correctly are not robustly degraded during translation, but rather through a relatively slow posttranslational process likely involving the cooperation of chaperones and degradation pathways (Vabulas and Hartl, 2005). In the timespan of 30–60 min, DRiP degradation might overlap with the regulated degradation of short-lived proteins (Vabulas and Hartl, 2005), and therefore the overall levels of erroneous protein synthesis remain undefined.

## 3 Co-translational degradation is a relatively rare but nonetheless relevant quality control mechanism

**FIGURE 1:**
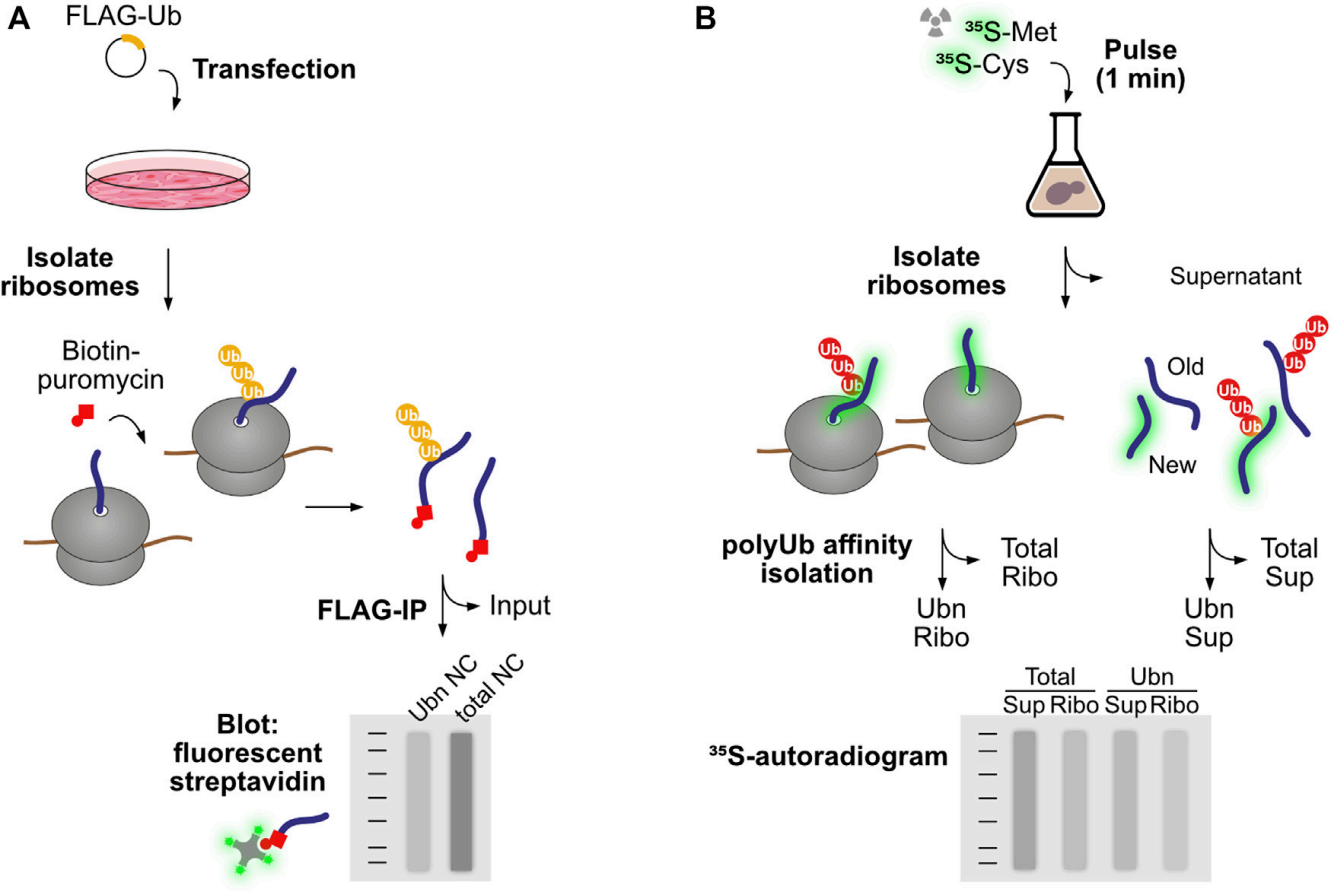
Experimental workflows to study co-translational ubiquitination. **(A)** Wang *et al.* introduced FLAG-tagged ubiquitin to human cells to enable purification of ubiquitinated protein species by FLAG immunoprecipitation. To restrict the analysis to nascent proteins, first ribosomes were isolated and associated polypeptides were labeled and released from the ribosome using biotin-puromycin. Following separation of ubiquitinated and non-modified species, nascent chains were detected based on the binding of fluorescent streptavidin with the biotin-puromycin label. **(B)** Duttler *et al.* pulse-labeled nascent proteins with ^35^S-labeled amino acids for a short period of time (1 min). After this pulse, some labeled nascent proteins were still associated with ribosomes, while others reached termination and ribosome release. Ribosomes were then isolated from completed proteins and both fractions were submitted to polyubiquitin affinity isolation. Nascent chains and newly synthesized released proteins were then detected by autoradiography. The fraction of co-translational ubiquitination is calculated as the amount of ^35^S-labeled nascent chains isolated by polyUb affinity over total ^35^S-labeled nascent chains in the ribosomal fraction.

These studies have raised very pertinent questions: to what extent do protein quality control pathways act co-translationally, and in which instances is co-translational degradation advantageous over engagement with the post-ribosomal chaperone network? Here we define co-translational degradation as all instances where nascent proteins are marked for degradation before their synthesis is completed, i.e., while still associated to ribosomes. Early studies observed co-translational ubiquitination of Apolipoprotein B, beta-galactosidase containing an artificial N-degron sequence, and *in vitro* translated CFTR (Sato et al., 1998; Zhou et al., 1998; Turner and Varshavsky, 2000). Wang *et al.* quantified overall levels of co-translational ubiquitination in human cells using a sensitive pulse labelling strategy (Figure 1A). Polysomes isolated from cells expressing FLAG-tagged ubiquitin were treated *in vitro* with biotin-conjugated puromycin, which incorporates into and releases nascent proteins from ribosomes. An anti-FLAG immunoprecipitation was used to isolate ubiquitinated proteins, which were then analyzed by SDS-PAGE and immunoblotting with fluorescent streptavidin to detect released nascent proteins. In HEK293T cells, 12%–15% of the total nascent polypeptides were found to be co-translationally ubiquitinated, with similar values also obtained in HeLa, mouse NIH 3T3, and primary human foreskin keratinocytes. Further analysis found that K48-linked chains were the predominant type of co-translational polyubiquitination, indicating a function in proteasomal degradation. The authors conclude that co-translational ubiquitination is a robust process in mammalian cells (Wang et al., 2013).

Duttler *et al.* performed similar analyses in yeast (Figure 1B). After a 1 min ^35^S-pulse labelling of nascent proteins, ribosome-bound nascent polypeptides were separated from finished products using sucrose fractionation, and both fractions were submitted to affinity isolation of polyubiquitinated proteins. The analysis showed that approximately 1.1% ± 0.07% of ribosome-bound nascent chains and 0.5% ± 0.04% of completed, newly made polypeptides were ubiquitinated *in vivo*. Chase experiments showed that these polyubiquitinated nascent polypeptides were rapidly degraded after 5–10 min, and proteasome inhibition increased the combined fraction of ubiquitinated nascent chains and newly made polypeptides from 1.5% to 5%. The authors conclude that a small pool of newly made proteins are ubiquitinated co- and post-translationally, and those are degraded during or soon after synthesis (Duttler et al., 2013). In this regard, it is important to point out that a steady flow of even a small percentage of ubiquitinated nascent proteins could represent a considerable burden to the proteasome, as ribosomes are 10 times more abundant than proteasomes in both yeast and mammalian cells (Russell et al., 1999; von der Haar, 2008; Beck et al., 2011; Duttler et al., 2013).

## 4 Types of co-translational degradation

Wang et al. (2013) observed that co-translational ubiquitination (CTU) can happen in at least two different contexts: within stalled translation complexes (CTU^S^) and within active translation complexes (CTU^A^). Ribosome run off experiments in human cells showed that CTU^A^ was the most prominent type of co-translational ubiquitination, representing circa two-thirds of CTU events. CTU^A^ increased in response to treatments that induce protein misfolding, such as the proline analog AZC, inhibition of co-translational chaperone Hsp70 (but not of post-translational chaperone Hsp90), or knockdown of the co-translational chaperone NAC (nascent polypeptide-associated complex). CTU^S^ was increased by agents that lead to translational errors or ribosomal stalling, such as Hygromycin B (affecting translational fidelity and readthrough of stop codons), Cycloheximide (inhibitor of ribosome translocation), and Eeyarestatin 1 (inhibitor of Sec61-dependent translocation).

## 5 The ribosome-associated quality control pathway: Targeting ribosome-stalled nascent chains for degradation

**FIGURE 2:**
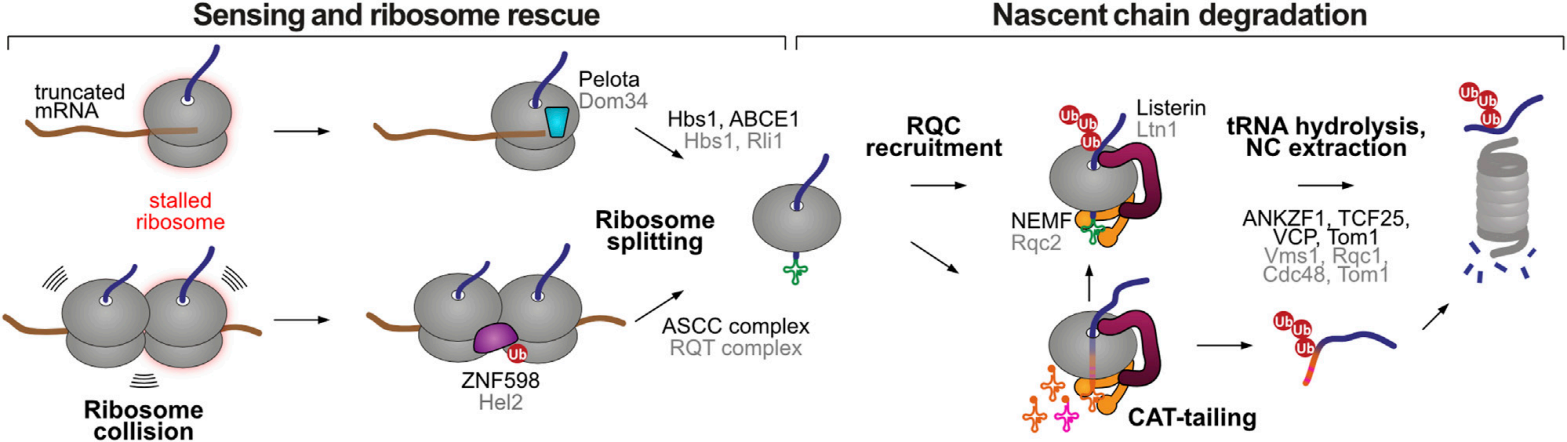
Stalled ribosome rescue and RQC pathways. Elongation-stalled ribosomes can be recognized by two mechanisms, an empty A site at the extreme 3′ end of the mRNA or the presence of collided ribosomes. Both situations lead to ribosome splitting, resulting in a 60S subunit still attached to the peptidyl-tRNA. The RQC marks the 60S-associated nascent chain for proteasomal degradation through two possible mechanisms, Listerin-mediated ubiquitination, or NEMF-mediated CAT-tailing. The latter can promote Listerin-mediated ubiquitination or act as a degron sequence after release from the large ribosomal subunit. Homologous human and yeast factors are labeled in black and grey, respectively.

While the effectors of CTU^A^ remain largely unknown, the major players of CTU^S^ have been extensively characterized in recent years. The function of the ribosome-associated quality control (RQC) pathway is to eliminate partially synthesized protein products from elongation-stalled ribosomes, i.e., ribosomes that take disproportionately long translational pauses and that are unable to undergo the canonical translation termination process. Initial sensing of stalled ribosomes can happen in two distinct ways (Figure 2): i) recognition of an empty ribosome A site (devoid of mRNA) by the protein Pelota (yeast Dom34), happening when ribosomes reach the extreme 3′ end of mRNAs lacking stop codons (Shoemaker et al., 2010; Pisareva et al., 2011; Hilal et al., 2016), or ii) recognition of collided ribosomes by the ubiquitin ligase ZNF598 (yeast Hel2), happening when a trailing ribosome encounters a leading ribosome that stalled mid-message (Juszkiewicz et al., 2018). In the first case, Pelota can induce ribosome splitting together with partner proteins Hbs1 and ABCE1. In the second case, ZNF598-mediated ubiquitination of specific sites at the 40S ribosomal subunit (in the RPS10 and RPS20 proteins) recruits the ASCC complex (named RQC-trigger complex, RQT, in yeast) to split the stalled ribosome (Juszkiewicz and Hegde, 2017; Matsuo et al., 2017; Sitron et al., 2017; Sundaramoorthy et al., 2017). In both instances, ribosomal splitting leads to a large ribosomal subunit still attached to the tRNA-bound nascent chain, a situation that does not occur in canonical translation and therefore serves as a signal for the recruitment of the RQC complex (Lyumkis et al., 2014). The core components of the RQC machinery in mammals are the ubiquitin ligase Listerin and the factor NEMF (Ltn1 and Rqc2 in yeast, respectively) (Chu et al., 2009; Bengtson and Joazeiro, 2010; Brandman et al., 2012). NEMF/Rqc2 recognizes the peptidyl−transfer RNA molecule at the interface of the dissociated 60S ribosome and supports the recruitment of Listerin/Ltn1 to ubiquitinate the stalled polypeptide near the ribosome exit tunnel (Shao et al., 2015). This is followed by tRNA release from the nascent chain by the tRNA endonuclease ANKZF1 (yeast Vms1) (Kuroha et al., 2018; Verma et al., 2018; Zurita Rendon et al., 2018), extraction from the 60S with the help of the AAA+ ATPase VCP (yeast Cdc48) and degradation by the ubiquitin-proteasome system, which also involves the factors TCF25 (yeast Rqc1) and Tom1 (Figure 2) (Brandman et al., 2012; Defenouillere et al., 2013; Verma et al., 2013; Defenouillere et al., 2017; Osuna et al., 2017; Kuroha et al., 2018).

In some situations, yeast Rqc2 and the large ribosomal subunit can elongate stalled proteins with carboxy-terminal alanine and threonine residues (CAT tails), in an atypical form of polypeptide elongation that does not involve a template mRNA or the 40S subunit (Shen et al., 2015). C-terminal extensions with varying amino acid composition have also been observed in prokaryotic and metazoan organisms, and therefore the term CAT tail has been revised to “C-terminal addition to translation tails” (Sitron and Brandman, 2020). CAT-tailing is thought to be important in instances where Listerin/Ltn1 fails to ubiquitinate the nascent chain due to the absence of Listerin-accessible Lys residues, i.e., when they are buried in the ribosomal exit tunnel or when they happen in inflexible structured regions (Kostova et al., 2017; Sitron and Brandman, 2019). CAT-tailing further extrudes the protein from the ribosomal exit tunnel, providing new opportunities for Listerin/Ltn1 ubiquitination (Kostova et al., 2017; Sitron and Brandman, 2019). In addition, the CAT tail can serve as a degron sequence, promoting Listerin/Ltn1-independent degradation of ribosome-stalled nascent chains. In the model cases studied so far, CAT-tailing promoted the recruitment of alternative E3 ubiquitin ligases (Hul5 in yeast and CRL2^KLHDC10^ and Pirh2/Rchy1 in humans) (Sitron and Brandman, 2019; Thrun et al., 2021) to polyubiquitinate the stalled nascent chains once they were released from the ribosome. Of note, CAT-tail recognition is predicted to happen exclusively off the ribosome, as CAT-tails (typically ∼10–14 residues (Sitron and Brandman, 2019)) would otherwise be buried inside the ribosomal exit tunnel.

Some aspects of CAT-tailed mediated protein degradation remain to be addressed: i) how are CAT-tailed proteins released from stalled ribosomes in the absence of Listerin/Ltn1, ii) are there additional ubiquitin ligases involved in CAT-tail induced degradation, and if so, are they specific towards certain substrates, iii) could CAT-tails have a direct function at the proteasome, for example in initiating protein unfolding and thus facilitating degradation (Thrun et al., 2021). In conclusion, RQC-mediated degradation can happen through two different co-translational mechanisms: ubiquitination by the Listerin/Ltn1 ubiquitin ligase, or CAT-tailing by the NEMF/Rqc2 factor. Accordingly, RQC-mediated degradation does not necessarily involve co-translational ubiquitination, with the CAT tail assuming the role of a degron. This mechanism might reconcile early observations that only a fraction (∼15%–18%) of ribosome-stalled nascent chains are ubiquitinated in human cells (Wang et al., 2013), and that Listerin/Ltn1 only accounts for a small fraction of total co-translational ubiquitination events (∼10% in human and ∼5% in yeast) (Duttler et al., 2013; Wang et al., 2013).

Importantly, CAT tails tend to self-associate, driving the formation of potentially toxic protein aggregates that sequester chaperone proteins and are difficult to eliminate (Choe et al., 2016; Defenouillere et al., 2016; Yonashiro et al., 2016; Wu et al., 2019; Sitron et al., 2020; Udagawa et al., 2021). This tendency was more pronounced for yeast CAT tails composed of Ala and Thr residues than for human CAT tails composed mainly of Ala residues (Yonashiro et al., 2016; Thrun et al., 2021; Udagawa et al., 2021). Disruption of Listerin/Ltn1 in yeast and human cells decreased stalled nascent chain degradation and promoted the accumulation of aggregated CAT-tailed proteins, sensitizing cells to translational and proteotoxic stress. NEMF/Rqc2 deletion ameliorated some of these phenotypes, indicating that CAT-tail accumulation contributes to cell toxicity (Choe et al., 2016; Defenouillere et al., 2016; Yonashiro et al., 2016; Wu et al., 2019; Sitron et al., 2020; Udagawa et al., 2021).

At the organismal level, mutations in the core RQC components have been associated with severe early onset motor and neurodegenerative phenotypes. In mice, a hypomorphic mutation in the gene encoding the Listerin ubiquitin ligase results in progressive neurological and motor dysfunction, which includes several pathological biomarkers seen in human neurological diseases (Chu et al., 2009). Mice harboring homozygous missense mutations in the *NEMF* gene also display a progressive neuromuscular phenotype (Martin et al., 2020). The corresponding mutations in yeast Rqc2 were associated with reduced CAT-tailing activity, but could still promote Ltn1 binding to stalled nascent chain complexes. NEMF-null mice exhibited more severe neuromuscular phenotypes than these hypomorphic mutants, having a median lifespan of 11 days (compared to 20 days and >2 years for the point mutants). NEMF mutations have also been found in nine patients presenting intellectual disability and/or early motor neuron disease phenotypes (Martin et al., 2020). Collectively, these observations indicate that CAT-tail induced protein aggregation can pose a significant risk to proteome balance, but (partial) loss of RQC-mediated protein degradation has severe impact on organismal fitness even in the absence of CAT-tail aggregation.

Stalling of ribosomes before they reach the correct termination codon results in the production of truncated proteins that are likely to be dysfunctional. Partially synthesized proteins could have problems in folding and maturation, or they might lack functional domains and therefore potentially cause dominant negative effects (Brandman et al., 2012; Brandman and Hegde, 2016). Some truncated proteins might be able to fold into a stable structure, and therefore a conformational criteria would not suffice to monitor all elongation-stalling events (Brandman and Hegde, 2016). Monitoring ribosome elongation status not only solves this problem, but also gives an opportunity to commit elongation-stalled nascent chains to degradation before they exit the ribosome. This prevents further attempts to fold these defective proteins, reducing the burden on the proteostasis network, and limits their chances to engage in inappropriate interactions. Moreover, adopting prolonged ribosome collision as a signal for RQC activation provides the opportunity to fine tune the RQC degradation threshold for different proteins: transcripts with low translation initiation rates have larger distances between ribosomes, allowing for transient translational pauses that serve a function in co-translational folding and trafficking (Juszkiewicz et al., 2018; Lakshminarayan et al., 2020).

## 6 Triggers of ribosomal stalling and RQC-mediated degradation

Since Listerin was first described to act in the degradation of elongation-stalled nascent chains, several situations were shown to cause ribosomal stalling and RQC activation. Many studies using model protein constructs provided proof-of-principle evidence that certain mRNA defects can activate the RQC. As RQC represents a proofreading pathway acting on relatively rare but very diverse failed translation events, obtaining a comprehensive view of endogenous RQC substrates is a challenging task that remains to be fully addressed. Characterizing the natural causes of RQC-mediated degradation, and how they respond to translational and proteotoxic stress, will be critical towards understanding how toxic protein species contribute to RQC-linked neurodegeneration (Sitron and Brandman, 2020).

### 6.1 Defective mRNAs

In yeast, the RQC is intimately linked to two cytosolic mRNA decay pathways that rely on ribosome translation as a proofreading mechanism to detect defective mRNAs: the No-Go Decay (NGD) pathway, which eliminates mRNAs that present obstacles to elongation (Doma and Parker, 2006; Simms et al., 2017; Ikeuchi et al., 2019; Winz et al., 2019), and the Non-Stop Decay (NSD) pathway, which targets mRNAs that lack stop codons (Frischmeyer et al., 2002; Pisareva et al., 2011; Tsuboi et al., 2012). As both pathways are activated by ribosomal stalling, they share common recruitment mechanisms with the RQC. Although the NGD and NSD pathways are conserved from yeast to human (Powers et al., 2020), experiments exploring whether related mRNA decay occurs in mammalian cell lines produced mixed results (Frischmeyer et al., 2002; Arthur et al., 2015; Juszkiewicz and Hegde, 2017; Meydan and Guydosh, 2020; Goldman et al., 2021). Mammalian cells minimize the translation of problematic messages *via* an mRNA silencing feedback loop. The protein EDF1 is a ZNF598-independent sensor of ribosome collisions that recruits the translational repressors GIGYF2 and EIF4E2 to prevent translation re-initiation at the defective mRNA, reducing the number of ribosomes queuing after the stalled ribosome and the protein output of the corresponding mRNA (Hickey et al., 2020; O'Donnell et al., 2020; Sinha et al., 2020).

mRNA defects that have been shown to cause a problem in ribosome elongation include:

#### 6.1.1 Premature polyadenylation

**FIGURE 3:**
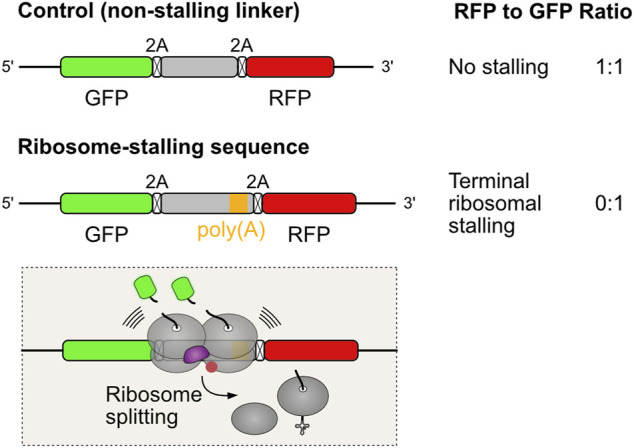
Dual fluorescence reporter assay for measuring terminal ribosomal stalling. Three coding sequences are expressed from the same mRNA, separated by viral 2A peptides that induce ribosomes to skip the formation of one peptide bond without interrupting translation (Lin et al., 2013). When translation proceeds undisturbed, the three proteins are produced in equal amounts. Problems in ribosome elongation during the synthesis of the test sequence lead to ribosome collision and recruitment of ZNF598/Hel2 to activate rescue of stalled ribosomes. Ribosome splitting, the commitment step in aborting translation, precludes the synthesis of RFP. This reflects in a reduction in the RFP to GFP fluorescence ratios, which can be quantified by flow cytometry analysis.

Alternative polyadenylation is a widespread mechanism of gene regulation that generates distinct mRNA 3′ ends to modulate mRNA localization, stability, and translation efficiency during developmental and signaling processes. This regulatory heterogeneity comes at the expense of fidelity. Premature polyadenylation is a naturally occurring error in transcript maturation where cleavage and polyadenylation happen at near cognate sites within the coding sequence (*i.e.* before the stop codon), rather than at the 3′ untranslated region (UTR). Unless a termination codon is created at the intersection of the coding sequence and the poly(A) sequence, ribosomes will read into the poly(A) tail. Using dual fluorescence reporter assays for terminal ribosome stalling (Figure 3), it has been shown that poly(A) sequences are strong inducers of ribosome stalling, but disruption of collision sensor ZNF598/Hel2 allows for eventual readthrough of such problematic sequences (Juszkiewicz and Hegde, 2017; Sundaramoorthy et al., 2017; Juszkiewicz et al., 2018). Translation of the first few AAA codons, encoding for Lys residues, progressively slows elongation as the poly(Lys) peptidyl tRNA adopts a conformation that is suboptimal for peptide bond formation. Furthermore, the poly(A) mRNA sequence inside the ribosome adopts a helical conformation that engages in stacking interactions with the 18S ribosomal RNA. These interactions promote rearrangement of the decoding center, further slowing elongation to the point of stalling (Chandrasekaran et al., 2019).

It has been postulated that premature polyadenylation is the most common mRNA defect in eukaryotes (Chandrasekaran et al., 2019). A comprehensive polyadenylation site database compiled from deep sequencing data estimate that 1.8% of the poly(A) sites mapped in human cells happen inside the coding sequence (Wang et al., 2018). However, these sites are used with very low frequency in relation to canonical 3′ UTR sites, and in some instances a stop codon might be formed. Of note, poly(A) site selection is regulated according to developmental and environmental conditions, and therefore fluctuations in premature polyadenylation mistakes might happen as well.

Another possibility for poly(A) translation is readthrough of stop codons, which could result in ribosomal stalling at poly(A) sequences correctly placed at the 3′ UTR. Although termination readthrough happens with some frequency (typically less than 1%), the majority of transcripts (93% in human cells) contain additional in frame stop codons within the 3′ UTR (Namy et al., 2001; Wangen and Green, 2020). Therefore, poly(A) translation would require consecutive rare readthrough events. Nevertheless, stop codon readthrough can be increased in certain situations, such as aminoglycoside antibiotic treatment (Palmer et al., 1979; Manuvakhova et al., 2000; Wangen and Green, 2020), stress-induced loss of the ABCE1 ribosome splitting factor (Annibaldis et al., 2020), and the [PSI+] prion state in yeast, involving aggregation of the Sup35p (eRF3 homolog) translation termination factor (Paushkin et al., 1996).

#### 6.1.2 Truncated mRNAs

Ribosomes stall at the extreme 3′ end of truncated mRNAs lacking stop codons, due to a simple physical impediment (Meaux and Van Hoof, 2006; Tsuboi et al., 2012; Guydosh and Green, 2014). Truncated mRNAs can arise, for example, by endonucleolytic cleavage within the open reading frame. Presumably, ribosomal stalling only happens when cleavage occurs in mRNAs already engaged in translation, as translation initiation is inefficient in the absence of a poly(A) tail (Munroe and Jacobson, 1990; Joazeiro, 2017). This could be the case during NSD and NGD decay pathways that cleave mRNAs at ribosome stalling sites, as an initial step in mRNA degradation (Frischmeyer et al., 2002; van Hoof et al., 2002; Doma and Parker, 2006; D'Orazio et al., 2019; Glover et al., 2020). Endonucleolytic cleavage of actively translated mRNAs might also happen in the context of regulated IRE1-dependent decay (RIDD), a process in which the IRE1 ribonuclease cleaves mRNAs located at the ER membrane in order to decrease the burden of ER protein synthesis during stress situations (Hollien and Weissman, 2006; Hollien et al., 2009). Additional cellular processes, such as nonsense-mediated mRNA decay (Huntzinger et al., 2008; Eberle et al., 2009) and some cases of gene silencing by microRNAs (Shin et al., 2010), also involve mRNA cleavage. Whether mRNA cleavage events in these different contexts activate the RQC remains to be demonstrated.

**FIGURE 4:**
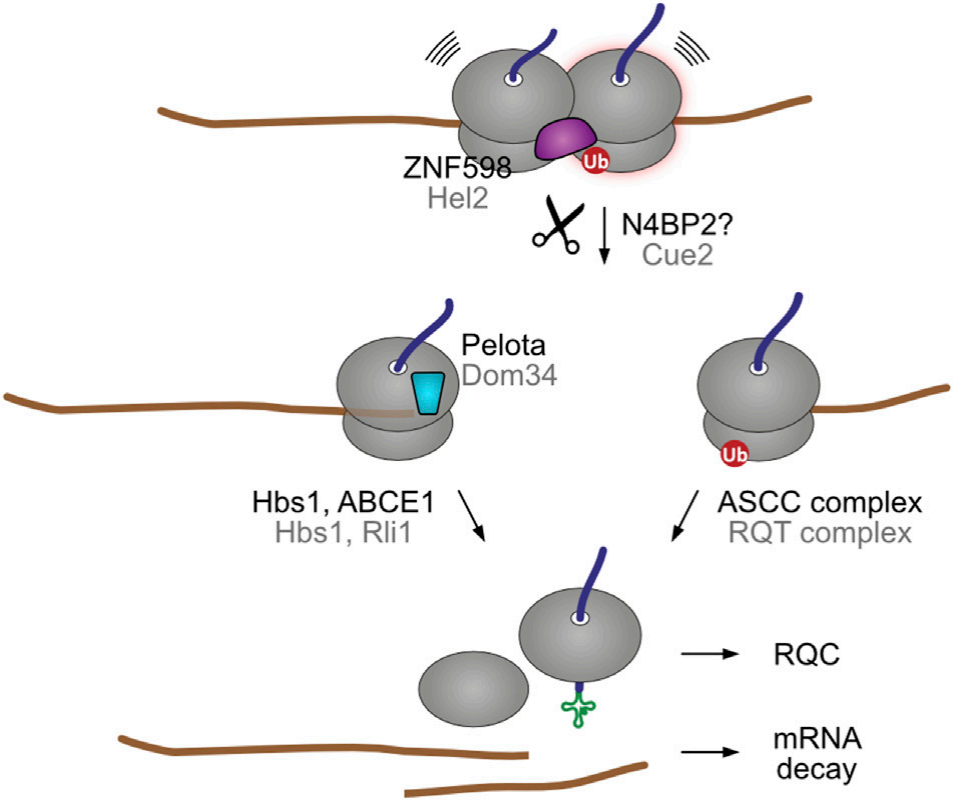
Endonucleolytic cleavage can result in secondary stalling. In yeast, nematodes, and possibly in humans, the ribosome collision sensor Hel2 recruits the Cue2 endonuclease (NONU-1 in worm) to cleave the encoding mRNA in the vicinity of the ribosome collision site. The ubiquitinated stalled ribosome is then split by the RQT ribosome rescue complex. The trailing ribosome subsequently stalls at the new 3′ end of the resulting truncated mRNA. This exposes an empty A site that is recognized by the Dom34 factor, which recruits the Hbs1-Rli1 factors for ribosomal splitting. Although humans have a Cue2 homolog (N4BP2), it is not yet clear if it engages in collision-dependent mRNA decay. Human and yeast homologs are labeled in black and grey, respectively.

One important implication from the eventual recruitment of mRNA decay pathways to collided ribosomes is that both stalling sensing mechanisms, ZNF598- and Pelota-mediated, could take place in the same mRNA (Figure 4). For example, ribosome collisions at a premature poly(A) site are initially recognized by the ZNF598/Hel2 collision sensor, which recruits the NGD pathway to cleave the problematic mRNA in the vicinity of the collision site (Simms et al., 2017; Ikeuchi et al., 2019; Winz et al., 2019). The next incoming ribosome then stalls at the new 3′ end, exposing an empty A site that is recognized by Pelota/Dom34. As stalled ribosome rescue is a relatively slow process (Goldman et al., 2021), it is also plausible that in truncated mRNAs additional ribosomes might collide after the 3′ end stalling event, thereby engaging ZNF598/Hel2. As ZNF598/Hel2 and Pelota/Dom34 recruit different ribosome splitting complexes, endonucleolytic cleavage might provide a fail-safe mechanism for stalled ribosome rescue.

#### 6.1.3 mRNA damage

Modification of mRNA can occur in certain physiological and stress situations, e.g., though reactive oxygen species (ROS), alkylating agents, or ultraviolet (UV) irradiation. *In vitro* translation using wheat germ extracts or rabbit reticulocyte lysates of messages containing the oxidised base lesion 8-oxoguanosine (8-oxoG) failed to produce a full-length protein (Tanaka et al., 2007; Simms et al., 2014). Instead, truncated peptide species were detected together with accumulation of peptidyl-tRNA (Simms et al., 2014). Yeast *in vivo* experiments showed that depletion of stalled ribosome rescue factor Dom34 increased the 8-oxoG level in poly(A) mRNAs in 80S and polysome fractions (Simms et al., 2014). Treatment with both the oxidizing agent 4-NQO and the alkylating agent MMS led to an increase in ubiquitination of nascent peptide chains (Yan et al., 2019). This effect was ablated upon Ltn1 depletion and increased upon Cdc48 knockdown, both findings indicating an activation of RQC (Yan et al., 2019). Furthermore, Hel2-mediated ribosome ubiquitination increased upon treatment with oxidizing and alkylating agents (Yan et al., 2019). In mammalian cells, a single 8-oxoG modification in the coding sequence led to twice as fast degradation of the mRNA than the same modification in the 3′ UTR (Yan et al., 2019).

Another important physiological stress, UV irradiation, has been described to chemically alter RNA as well as DNA, e.g., with the formation of pyrimidine dimers (Kalthoff et al., 1978; Wurtmann and Wolin, 2009). Wu *et al.* hypothesized that UV irradiation not only stresses cells by damaging DNA and ribosomal RNA (rRNA) but also leads to mRNA damage resulting in ribosome collisions (Wu et al., 2020). Polysome profiling of UV-irradiated HeLa cells showed an increase in collided ribosomes compared to untreated cells (Wu et al., 2020; Stoneley et al., 2022). Codons with two adjacent pyrimidines were especially associated with collided ribosomes, indicating that stalling happens at sites particularly susceptible to UV damage (Wu et al., 2020). Further evidence was provided recently, when *in vitro* translation of a UV irradiated mRNA harboring a pyrimidine tract led to formation of tRNA-bound proteins truncated at the pyrimidine site, indicating that mRNA damage is sufficient to induce ribosomal stalling (*i.e.* in the absence of rRNA and tRNA lesions) (Stoneley et al., 2022). Interestingly, Stoneley et al. (2022) found that the cause of stall influences its outcome: elongation inhibitor-, UVB- and 4NQO-stalled ribosomes all recruit the ASCC ribosome splitting complex, but it fails to resolve the stall for UVB- and 4NQO-stalled ribosomes. Combined, these studies demonstrate that mRNA damage caused by alkylating or oxidizing agents or UV irradiation hinder translation and result in ribosome collisions. Considering that the abundance of oxidized RNA is correlated with various neurodegenerative diseases (Nunomura et al., 2006), ribosome-associated quality control processes are likely to play an important role in attenuating disease-related toxic effects. Nonetheless, open questions remain, such as the mechanisms by which mRNA modifications interfere with ribosomal elongation, and to what extent UV-induced aminoacyl-tRNA and rRNA damages hinder decoding properties during translation.

### 6.2 Inefficient translation termination

Besides problems in ribosome elongation, also inefficient translation termination can lead to ribosome collisions and RQC activation. Wu *et al.* report that in a *Drosophila* genetic model of Parkinson’s disease and in human cells treated with a mitochondrial stressor, the mitochondrial C-I30 protein (Complex I 30kD protein, a nuclear-encoded part of the C-I respiratory chain complex) is extended with CAT-tails at the stop codon, leading to its aggregation. The authors named this process MISTERMINATE (mitochondrial-stress-induced translational termination impairment and protein carboxyl terminal extension). Overexpression of translation termination factors, ABCE1 and eRF1, reduced NEMF-mediated C-I30 extension, indicating that MISTERMINATE is caused by an insufficiency of translation termination factors (Wu et al., 2019). The activity of the essential ribosome splitting factor ABCE1 (yeast Rli1), involved in both canonical translation termination and Pelota-activated stalled ribosome rescue, depends on oxidation-labile Fe-S clusters present at its N-terminal domain (Barthelme et al., 2007). The initial reactions that assemble Fe-S clusters on protein scaffolds occur in the mitochondrial matrix, and therefore ABCE1 synthesis requires proper mitochondrial fitness (membrane potential and active transport) (Lill and Muhlenhoff, 2008). Consequently, maintenance of ABCE1/Rli1 protein abundance is compromised during diverse oxidative and mitochondrial stress situations (Alhebshi et al., 2012; Sudmant et al., 2018; Wu et al., 2019; Zhu et al., 2020). Ribosome profiling studies showed that a decrease in ABCE1/Rli1 levels are associated with widespread accumulation of ribosomes at the stop codon and at the 3′ UTR, consistent with ribosomal collisions and stop codon readthrough (Young et al., 2015; Mills et al., 2016; Sudmant et al., 2018). To what extent a termination dysfunction activates RQC-mediated degradation in prolonged situations of stress remains to be defined.

### 6.3 Charged tRNA insufficiency

Codon usage can influence translation kinetics during protein synthesis, and this phenomenon is largely dependent on the abundance of cognate tRNAs (dos Reis et al., 2004; Pechmann and Frydman, 2013; Yu et al., 2015). Adjustment of local ribosomal translation speed mediated by alteration of optimal and non-optimal codons allows for enhanced co-translational protein folding (Zhang and Ignatova, 2011; Zhou et al., 2013). For example, a synonymous single nucleotide polymorphism in the gene encoding the polytopic membrane protein CFTR (T2562G) modifies local translation speed and alters CFTR stability (Kirchner et al., 2017). The stability decrease is rescued upon increasing the level of the corresponding tRNA (Kirchner et al., 2017). Charged tRNA availability can largely vary depending on tissue or even cell type. In some instances, insufficient supply of charged tRNAs can slow translation to the extent of provoking ribosome collisions.

Mutated glycyl-tRNA synthetase (GARS), causing Charcot-Marie-Tooth type 2D (CMT2D) peripheral neuropathy, fails to release charged tRNA^Gly^ (Mendonsa et al., 2021; Zuko et al., 2021). Sequestration of tRNA^Gly^ from the cellular pool depletes it for translation. Ribosomal profiling of HEK293T cells expressing a CMT-GARS mutant (G240R), as well as spinal cord extracts of CMT2D mice (*Gars* ^*C201R/+*^), showed accumulation of Gly codons in the ribosomal A site, indicating a prolonged dwell time on these codons (Mendonsa et al., 2021; Zuko et al., 2021). Depletion of GTPBP2, a mammalian Hbs1 homolog and binding partner of Pelota, worsened the peripheral neuropathy phenotype in *Gars* ^*C201R/+*^ mice, whereas it did not induce a phenotype by itself (Zuko et al., 2021). Similarly, loss-of-function mutation in n-Tr20, encoding for the CNS-exclusive tRNA^Arg^_UCU_, induces ribosome stalling at AGA codons in a mouse model (Terrey et al., 2020). Failure to resolve these stalling events occurs upon simultaneous impairment of GTPBP2 and consequently leads to neurodegeneration (Terrey et al., 2020). Of note, the neurodegenerative phenotype caused by loss of GTPBP2 could not be rescued by Hbs1l, another Pelota binding partner (Terrey et al., 2020). In conclusion, depletion of charged tRNAs from the cellular pool, either by a defective tRNA synthetase or mutations in the tRNAs themselves, promotes ribosome stalling events and can contribute to the pathogenesis of neurodegenerative disease.

### 6.4 Viral infection

Viruses hijack the host protein synthesis apparatus to produce viral proteins, having evolved remarkable strategies to manipulate the translation machinery to favor viral mRNAs. In response, cells attempt to limit infection by downregulating translation and activating a number of signaling pathways. Different lines of evidence indicate that viral infections increase the incidence of ribosomal collisions. ZNF598 knockdown suppressed poxvirus spread and viral protein synthesis (DiGiuseppe et al., 2018). Similarly, vaccinia virus replication was repressed more than 10-fold in both ZNF598-KO and eS10 or uS10 point mutant cell lines lacking the critical ZNF598 ubiquitin-acceptor residues (Sundaramoorthy et al., 2021). In wild-type (WT) cells, vaccinia virus increased uS10 ubiquitination throughout the infection. Proteomic and transcriptomic analyses indicated that ZNF598 disruption affects primarily viral protein synthesis, rather than transcription (Sundaramoorthy et al., 2021). These observations suggest that vaccinia virus infection enhances the frequency of ribosome collisions and that an inability to rescue stalled ribosomes limits viral translation, presumably by depleting the pool of functional ribosomes (Sundaramoorthy et al., 2021). Of note is that vaccinia virus inhibits the integrated stress response (ISR), which normally would reduce protein synthesis in situations of proteotoxic stress (Davies et al., 1992; Davies et al., 1993). Accordingly, reprogramming cells to sustaining high translation rates despite the elevated proteostasis burden likely increases viral dependency on the stalled ribosome rescue system (Sundaramoorthy et al., 2021).

Ribosome collisions also play a role in immune signaling. Depletion of ZNF598 or ASCC3 (a helicase mediating stalled ribosome splitting) activated interferon-stimulated gene (ISG) expression, which induces a broad antiviral state (Li et al., 2013; Williamson et al., 2017; DiGiuseppe et al., 2018). The cyclic GMP-AMP synthase-stimulator of interferon genes (cGAS-STING) pathway plays a vital role in triggering the innate immune response, by sensing cytosolic DNA as an indication of invading pathogens. A recent study has shown that translation stress acts as a potent co-activator of this pathway (Wan et al., 2021). cGAS was found to interact with collided ribosomes, and chemical induction of ribosomal collisions increased cGAS relocation from the nucleus to ribosomes in the cytosol, where it activated downstream signaling pathways culminating in ISG expression (Wan et al., 2021). The same happened when collided ribosomes accumulated as a result of ZNF598 or ASCC3 inactivation. Of note, poxvirus and vaccinia virus replication also decreased upon ZNF598 depletion in cells lacking an interferon response, indicating that the function of ZNF598 in supporting protein synthesis also plays an important role in viral spread (DiGiuseppe et al., 2018; Sundaramoorthy et al., 2021). While RQC-mediated degradation of viral proteins remains to be formally demonstrated, collectively these observations reveal that viral infections promote a state of collision-inducing translational stress, and that this state is exploited by the immune system to detect intracellular infections. Besides the role in ISG activation, the RQC could also contribute to timely sampling of viral proteins for MHC-I antigen presentation, given its ability to rapidly target a wide range of proteins independently of their folding properties (Trentini et al., 2020).

### 6.5 Failed membrane insertion

**FIGURE 5:**
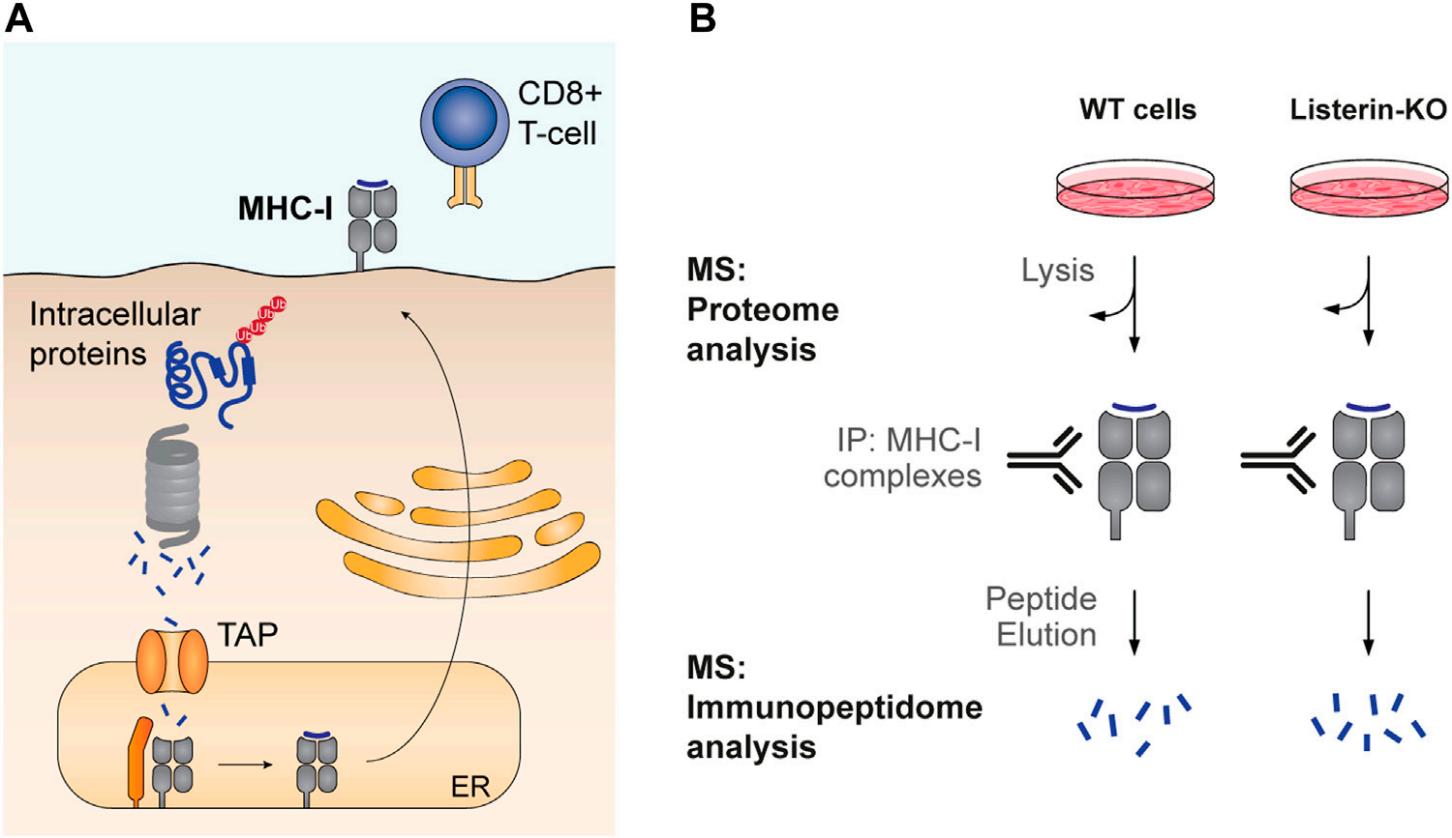
Analysis of MHC-I antigen presentation as a proxy for protein degradation. **(A)** Cytosolic peptides produced by the proteasome are translocated into the ER lumen by the Transporter associated with Antigen Processing (TAP). There, they are loaded into the MHC-I complex, which is then transported to the cell surface for inspection by cytotoxic T cells. Accordingly, MHC-I presentation correlates with the levels of intracellular peptides produced by proteasomal degradation. **(B)** High-throughput analysis of MHC-I antigen presentation. Trentini *et al.* quantified the repertoire of immunopeptides in WT and Listerin-KO human cells by immunoprecipitation of MHC-I with bound peptides. Peptides were released from the complex by solvent elution and then analyzed by mass spectrometry (MS). An aliquot of the total protein extract was collected for trypsin digestion and total proteome analysis. Comparison between proteome and immunopeptidome WT to KO ratios allows assessing whether changes in antigen presentation stem from differential protein expression or from alterations in proteasomal degradation.

An inventory of natural targets of Listerin-mediated degradation in human cells has been recently obtained by serendipitous methods. Prompted by a long-standing debate concerning how defective protein synthesis supports antigen presentation, Trentini *et al.* analyzed the contribution of the Listerin ubiquitin ligase to MHC-I peptide repertoire in human cells (Trentini et al., 2020). MHC-I antigen presentation involves the loading of cytosolic peptides produced by the proteasome system into the MHC-I protein complex, followed by their transport to the cell surface for inspection by cytotoxic T-cells (Figure 5A). This process allows the immune system to detect intracellular pathogens and cancer-transformed cells. As MHC-I antigen presentation can be used as a proxy for proteasomal degradation, mass spectrometry based immunopeptidome analysis of WT and Listerin-KO cells revealed how Listerin deletion affects the degradation of endogenous human proteins (Figure 5B). Overall, the analysis identified a total of 3,658 immunopeptides belonging to 2,016 different proteins. Of those, 103 peptides from 100 different proteins (∼3% of the detected immunopeptidome and ∼5% of presented proteins) were significantly more abundant in WT cells than in Listerin-KO cells, and therefore represent targets of RQC-mediated degradation. Gene ontology enrichment analysis did not show any significant differences between the group of Listerin targets and the entire list of presented proteins, indicating that RQC-mediated degradation is not biased towards a specific molecular function, cellular component, or biological process (Trentini et al., 2020).

Further indicators of the physiological role of RQC came from total proteome analysis of WT and Listerin-KO cells. Interestingly, RQC disruption caused an accumulation of subunits of the ER membrane protein complex (EMC) and the translocase of the outer membrane (TOM) (Trentini et al., 2020). The EMC and TOM complexes are implicated in the insertion of transmembrane proteins into the ER and mitochondria, respectively. TOM forms the mitochondria entry gate for most nuclear-encoded mitochondrial proteins, and both post-translational and co-translational import mechanisms have been reported (den Brave et al., 2021). EMC is a widely expressed and highly abundant 10 subunit protein complex that can act as a co-translational membrane insertase for specific protein topologies and as a potential chaperone for transmembrane segments (Chitwood et al., 2018; Guna et al., 2018; Shurtleff et al., 2018; Miller-Vedam et al., 2020). *In vivo* characterization of EMC substrates revealed a preference for multipass membrane proteins and for proteins containing charged residues within transmembrane domains (TMDs) (Shurtleff et al., 2018; Tian et al., 2019). The Listerin-KO immunopeptidome data indicated that transmembrane proteins in general (number of TMDs ≥1) were not overrepresented among Listerin targets (Trentini et al., 2020). However, there was a significant enrichment of proteins with large numbers (>10) of TMDs: 4% of the Listerin targets *versus* 1.5% among proteins with Listerin-independent presentation. Moreover, the WT/KO immunopeptidome ratio (*i.e.* the effect of Listerin on presentation/degradation) increased with the number of TMDs, and proteins with more than 10 TMDs displayed significantly higher RQC-dependent degradation than proteins not having any predicted TMD. Taken together, the results indicate that complex membrane proteins have an increased tendency towards RQC-mediated degradation.

In theory, ribosome stalling during membrane insertion/translocation could be triggered by the same mRNA defects engaging the RQC pathway in the cytosol (von der Malsburg et al., 2015). Another possibility is cleavage of ER-localized mRNAs by the Ire1 ribonuclease during RIDD. Importantly, membrane proteins with low numbers of TMDs, secretory, or ER-resident proteins were not overrepresented among RQC targets in the reported immunopeptidome analysis (Trentini et al., 2020), hinting that translation at the ER was not associated with an increased risk of mRNA damage. The findings indicate that the intrinsic challenges of co-translationally inserting TMDs into the membrane render complex transmembrane proteins prone to RQC-mediated degradation. We therefore proposed that improper membrane insertion could result in stalling of translocon-anchored ribosomes, triggering RQC recruitment (Trentini et al., 2020).

This hypothesis has been further corroborated by a study in yeast addressing the biogenesis of the ABC transporter Yor1. Lakshminarayan et al. (2020) report that the synthesis of Yor1-ΔF_670_ misfolded mutant is further compromised by disruption of the EMC insertase/chaperone complex, without major changes to global fold or aggregation state of the Yor1-ΔF_670_ nascent protein. The synthesis defect caused by EMC loss was reversed by the deletion of genes acting on RQC recruitment: Hel2, Dom34, and the RQT complex subunit Slh1 (ASCC3 homolog). As these gene deletions allow for read-through of translation stalls, it was proposed that the combined effects of the ΔF_670_ folding defect and the loss of EMC activity exacerbate ribosomal pausing during Yor1 synthesis, leading to ribosome collisions. A similar observation was also made for a Sec61 translocon mutant (R275E/R406E) impaired in co-translational targeting: Hel2 deletion reversed the detrimental effect of this Sec61 mutation on the synthesis of Yor1-ΔF_670_. The results indicate that a ribosome-associated quality-control pathway is recruited by ribosome collisions when transmembrane domain insertion and/or folding fails. Whether this pathway involves the canonical RQC components remains to be tested. The authors also show that yeast mRNAs encoding for polytopic membrane proteins have relatively low translation efficiency (ribosome abundance along the mRNA), suggesting an evolutionary adaptation to permit ribosome speed reductions required for productive transmembrane protein synthesis and maturation (Lakshminarayan et al., 2020).

These two studies support the emerging concept that, at least in the context of co-translational membrane insertion, ribosome stalling and RQC activation may not originate from mRNA defects. Another novel implication is that, as the mechanisms of RQC activation perceive ribosome elongation status and not the folding state of the nascent chain (Trentini et al., 2020), problems in transmembrane protein folding/insertion can influence ribosome elongation. The exact mechanisms leading to ribosomal stalling and RQC activation during the synthesis of complex membrane proteins remain to be characterized, and are likely to differ from those at the cytoplasm (Phillips and Miller, 2020). Lakshminarayan *et al.* posit that Yor1 TMDs might engage in hydrophobic interactions with the ribosomal exit tunnel, which could create kinked nascent chain structures that impair elongation, akin to what has been observed for translation arrests induced by the drug-like molecule PF846 (Li et al., 2019; Lakshminarayan et al., 2020). These transient stalls could be counteracted by pulling forces provided by correct folding and/or chaperone activity, similar to the mechanisms that relieve bacterial stalling signals (Wilson et al., 2016; Lakshminarayan et al., 2020). Indeed, it is plausible that the peculiar characteristics of TMDs, high hydrophobicity and the tendency to adopt α-helical conformation inside the ribosomal tunnel (Bano-Polo et al., 2018), pose a challenge for their translocation across the ribosome exit tunnel. How problematic TMDs interact with the ribosomal exit tunnel, and whether they can distort the peptidyl-transferase center to the point of compromising ribosome elongation, remain to be addressed. Nevertheless, it has been experimentally shown that, in bacteria, interactions between nascent polypeptides and lipids can provide pulling forces for TMD partitioning from the translocon into the membrane, and that intramolecular interactions between N- and C-terminal TMDs can increase these pulling forces (Cymer and von Heijne, 2013; Cymer et al., 2014; Niesen et al., 2018). These observations provide a rationale for how correct folding and the activity of the translocon and its associated factors can overcome the intrinsic challenges of translating transmembrane proteins.

An alternative possibility is that obstacles in TMD access to the membrane pose a steric hindrance to the elongation activity of translocon-bound ribosomes. In principle, physical roadblocks preventing access of substrates to the membrane could arise from defective/mutated membrane insertion machinery (translocon and its associated factors) (Phillips and Miller, 2020), or from inappropriate nascent chain conformations in the vicinity of the membrane access site. Recent studies reveal that biogenesis of multipass membrane proteins involves substrate-driven, dynamic assembly of multiple factors to the ribosome-Sec61 complex: the GET- and EMC-like (GEL), protein associated with translocon (PAT), and back of Sec61 (BOS) complexes (referred as the “multipass translocon”) (Smalinskaite et al., 2022; Sundaram et al., 2022). These findings underscore the high complexity of co-translational folding/insertion of large transmembrane proteins, which provides ample opportunity for error. Of note, many transmembrane proteins contain charged and polar residues within membrane-spanning segments that are structurally and functionally important, for example, in forming hydrogen bonds or salt bridges between TMDs, and in establishing conductance channels and membrane occlusion sites of transporter proteins (Adamian and Liang, 2002; Partridge et al., 2002). As a result, certain TMDs are only marginally hydrophobic, and thus, are difficult to partition into the lipid bilayer. Interestingly, all five multipass membrane proteins characterized as RQC targets by immunopeptidome analysis belong to the solute carrier (SLC) family of membrane transport proteins (Trentini et al., 2020), which commonly employ charged residues in membrane-spanning segments. This observation hints that particularly challenging TMD insertion events might promote ribosome collisions and RQC activation.

Another particular problem of membrane protein biogenesis is establishing the correct topology (*i.e.* orientation in relation to the membrane plane). In the case of polytopic membrane proteins, the first TMD is considered critical for setting the overall topology in which downstream TMDs will be placed (Blobel, 1980). Topology mistakes can occur, for example, when EMC activity is lacking. The EMC complex is necessary for establishing the correct orientation of multipass proteins adopting an exoplasmic N-terminus conformation, by mediating the insertion of the first TMD sequence in this conformation (Chitwood et al., 2018). It is unlikely that a wrong orientation arising from insufficient EMC activity represents the mechanism responsible for stalled Yor1-ΔF_670_ translation in EMC deletion strains, or for RQC-mediated degradation of the five SLC proteins identified by Trentini *et al.*, as all these proteins assume an endoplasmic N-terminus topology. Nevertheless, in theory topology mistakes could hamper co-translational nascent chain folding/insertion, by limiting appropriate access to luminal and cytoplasmic chaperone and assembly factors.

**FIGURE 6:**
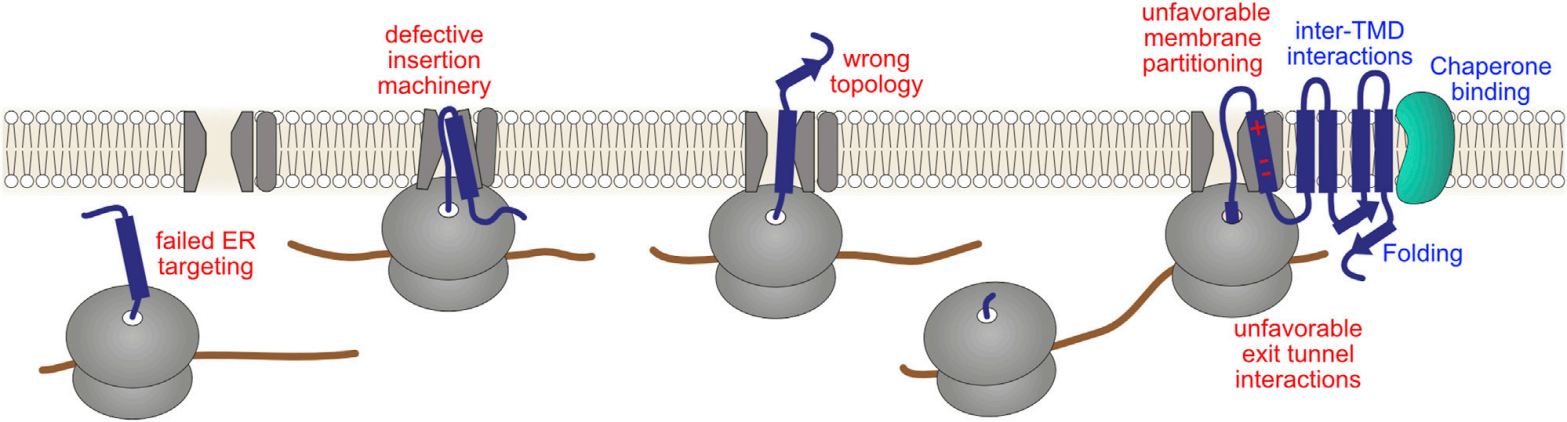
Challenges in complex transmembrane protein biogenesis. Incorporation of proteins into the membrane entails overcoming important biophysical challenges: hydrophobic protein segments must be shielded from the crowded aqueous cytosol as well as transported past the highly polar surface of the membrane into the hydrophobic core of the lipid bilayer. Therefore, the majority of transmembrane proteins are assembled co-translationally at the ER. This entails targeting of ribosomes to the Sec61 translocon, defining the protein topology (orientation in relation to the membrane plane), inserting TMDs into the membrane *via* concerted action of the translocon and additional folding/insertase complexes, translocation of soluble luminal domains, initial stages of folding, and in some cases glycosylation of the nascent chain. Potential failures in these co-translational processes (red labels), either due to defective cellular machinery or a defective nascent chain, might in principle affect ribosomal elongation. Productive maturation steps, such as folding of soluble domains, formation of correct inter-TMD interactions and inter-domain assembly, and chaperone-mediated remodeling (blue labels), might aid ribosomes in overcoming these problems.

In conclusion, critical challenges of multipass transmembrane protein biogenesis–targeting ribosomes to the ER, defining membrane topology, TMD insertion into the lipid bilayer, and some aspects of folding–have to be addressed co-translationally (Figure 6). Understanding to what extent and how malfunctions in these different steps, either due to nascent chain mutations or natural and stress-induced deficits in the activity of biosynthesis factors, compromise ribosome elongation and activate co-translational degradation represents a key area for further investigation.

### 6.6 Failed ribosome recruitment to the ER membrane

Co-translational targeting of proteins to the ER is generally mediated by the signal recognition particle (SRP), which recognizes hydrophobic segments (signal sequences or TMDs) of nascent polypeptide chains and, through interaction with the ER-localized SRP receptor, directs them to the translocon. This process involves transient translation arrest until the SRP-bound ribosome–nascent chain complex (RNC) is delivered to the SRP receptor, which is required to maximize ER targeting (Walter and Blobel, 1981; Mason et al., 2000; Lakkaraju et al., 2008). It would be counterproductive if such programmed translation arrests activated the RQC and/or No-Go mRNA decay. The mechanisms preventing this from happening remain to be fully understood.

A recent study used affinity purification of yeast Hel2-bound RNCs followed by sequencing of ribosome-protected mRNA fragments to characterize endogenous substrates of Hel2. Matsuo and Inada observed that Hel2-associated cytosolic ribosomes are enriched for mRNAs containing a signal sequence and/or TMD(s). This enrichment was lost when both cytosolic and ER-localized ribosomes were included in the analysis, indicating that Hel2 frequently targeted secretory RNCs before they engage with the Sec61 translocon. The association of Hel2 with cytosolic ribosomes translating secretory proteins was increased by reducing the expression of SRP, suggesting that Hel2 targets ribosomes lacking SRP recognition. Of note, SRP depletion increases mistargeting of secretory proteins to the mitochondria and rapidly induces mitochondrial fragmentation (Costa et al., 2018). Deletion of Hel2 in the low SRP expression yeast strain was associated with mistargeting of the ER membrane protein Sct1 to mitochondria and with a growth reduction in respiratory medium (Matsuo and Inada, 2021). The authors propose that Hel2 participates in a triage system that detects secretory RNCs lacking SRP recognition and prevents them from mislocalizing to mitochondria. Further mechanistic studies will be required to clarify the role of Hel2 in this putative quality control system of the secretory pathway.

ER targeting involves the concerted interplay between SRP and the co-translational chaperone NAC (nascent polypeptide–associated complex) (Wiedmann et al., 1994). The abundant NAC complex is capable of binding both SRP and the ribosome simultaneously, increasing the local concentration of SRP in the vicinity of the ribosomal exit tunnel. However, NAC initially precludes direct SRP binding to the ribosome until an ER signal sequence emerges, upon which NAC rearrangement allows for SRP interaction with the nascent chain (Jomaa et al., 2022). This mechanism illustrates how protein targeting is accomplished through a multilayered process involving competition between multiple factors recruited to the ribosomal exit tunnel region.

Immunoprecipitation experiments of the Eukaryotic translation initiation factor 3 (eIF3) from fission yeast demonstrated that eIF3 assembles into a large supercomplex, coined the “translatome”, containing elongation factors, tRNA synthetases, 40S and 60S ribosomal proteins, chaperones, and the proteasome (Sha et al., 2009). A subsequent study in human cells demonstrated that, in addition to its function in translation initiation, eIF3 is capable of associating with 80S ribosomes translating the first ∼60 codons of a subset of mRNAs (Lin et al., 2020). Ribosome profiling experiments showed that knockdown of eIF3 subunit “e” resulted in increased association of ribosomes with a subset of mRNAs in the region between codons 25 and 75. Affected transcripts encompassed a large number of membrane proteins, as well as mitochondrial, endosomal, lysosomal, and secretory proteins. The observed increase in ribosome association correlated with a reduction in protein synthesis. The findings indicate that, for specific mRNAs, eIF3 deficiency causes a slow-down in early translation elongation, in a region that approximately coincides with the emergence of nascent chains from the ribosomal exit tunnel. The authors propose that one of the functions of eIF3 is to recruit factors to the 80S ribosome that receive nascent chains to target them to their subcellular destinations, which in turn promotes ribosome elongation (Lin et al., 2020). As proteasomes are also part of the eIF3 supercomplex, it is plausible that the translatome functions in ensuring translational fidelity in the early synthesis steps of a specific set of proteins, most notably transmembrane, secretory and mitochondrial proteins, by bridging protein synthesis and degradation machineries.

In conclusion, while transient translation arrest serves an important physiological function in targeting of nascent chains to the ER, failure in achieving the correct destination can also result in stalling of ribosomes in the early stages of translation. How cells distinguish between these two scenarios warrants further investigation, and is likely to involve the dynamic interplay between multiple ribosome-associated proteostasis factors.

## 7 Ribosome profiling experiments reveal general principles of ribosomal stalling

**FIGURE 7:**
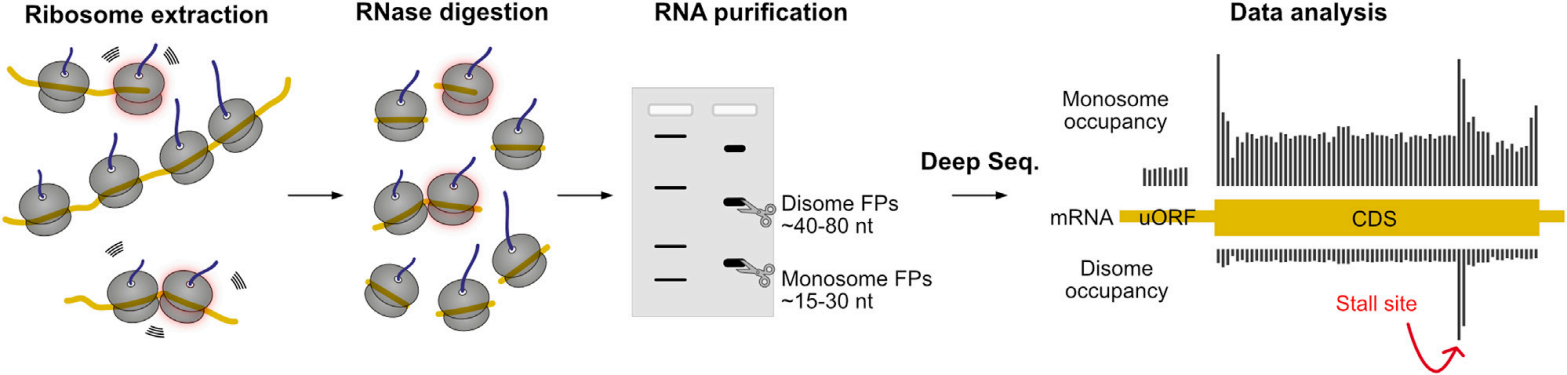
Ribosome profiling allows large-scale monitoring of translation dynamics. First, ribosome nascent-chain complexes are extracted from cells or tissues, and then they are treated with RNase. Translating ribosomes protect small pieces of mRNA from digestion, resulting in ribosome footprints (FPs) that reflect the position of the ribosome on the mRNA. The FPs of monosomes or stacked ribosomes are then isolated by electrophoresis and analyzed by deep sequencing technology. The abundance of monosome FPs serves as a quantitative measure of translation (ribosome association) at codon resolution. Disome footprints occur upon ribosomal stalling with subsequent ribosome collision, and are indicative of hard to decode sequences. The protocol can also be adapted to identify short footprints resulting from ribosomal stalling in the 3′ end of truncated mRNAs.

Ribosome profiling (also called Ribo-Seq or ribosomal footprinting) employs deep sequencing of ribosome-protected mRNA fragments to measure the position-specific association of translating ribosomes with mRNA (Ingolia et al., 2009). For this analysis, cell lysates are treated with a ribonuclease, ideally destroying all RNA except short mRNA regions protected by ribosomes, called ribosome footprints (RFPs) (Figure 7). Sequencing these ∼28-nucleotide oligomers offers a global snapshot of translation, and allows for a wide variety of analyses: determination of open-reading frame (ORF) boundaries–including upstream ORFs, non-ATG or alternative translation start sites, transcriptome-wide determination of translation rates, and quantification of changes in the translatome upon stress induction, drug treatment, or throughout different tissues, to name just a few. Furthermore, the ribosome occupancy on a single message allows estimating changes in local decoding speed: the number of ribosome-protected footprints of a specific site correlates with the relative dwell time of the ribosome, revealing hard-to-decode motifs, pause and stall sites (Ingolia et al., 2011). Following Guydosh and Green (2014), studies additionally sequenced footprints protected by disomes (*i.e*. two collided ribosomes, 40–80 nt) or even trisomes (80–100 nt), giving a clearer idea on which sequences ribosomes pause and begin to pile up. Moreover, ribosomes stalled at the 3′ end of truncated mRNAs can be identified as short (15–18 nt) footprints (Guydosh and Green, 2014). Han *et al.* (2020) identified over 2,200 disome sites in ∼1,100 individual genes in a genome-wide collision study in HEK293 cells, suggesting that ∼11% of all genes have at least one ribosome collision site (Han et al., 2020). *In vivo* profiling data of mouse liver suggest that ∼10% of elongating ribosomes are trapped in a disome state (Arpat et al., 2020). It has been previously hypothesized that collisions are more likely to occur on highly translated mRNAs due to denser packing of ribosomes (Juszkiewicz et al., 2018). Comparison of the translation efficiency (ratio of monosome reads to RNA-seq counts) to the rate of collision on the global scale gives contradicting results: no correlation was observed in yeast (Meydan and Guydosh, 2020) or mammalian cells (Han et al., 2020), whereas a strong positive correlation was found in mouse liver (Arpat et al., 2020).

Ribosome profiling studies have provided important insights into the determinants of stalled translation, revealing, for example, overrepresentation of amino acid motifs at collision sites. Mouse liver profiling showed a selectivity for amino acids only in the P- and A-sites of the leading ribosome: Asp was over-represented in both P- and A-site, Ile in the A-site and Gly in the P-site only (Arpat et al., 2020). Furthermore, enrichment of negatively (Asp, Glu) and depletion of positively charged amino acids (Lys, His) was observed (Arpat et al., 2020). A dependency on codon usage was only found in selected cases: Asp in the P-site only occurred when encoded by AAT, Lys was either enriched or depleted in the A-site when encoded by AAG or AAA, respectively (Arpat et al., 2020). Dipeptide motifs Gly-Ile, Asp-Ile and Gly-Asp were strongly enriched on pause sites independently of codon usage (Arpat et al., 2020). Several other dipeptide motifs were strongly dependent on codon usage, namely when Lys or Gly reside in the A-site (Arpat et al., 2020). Other studies identified Pro-Pro/Gly/Asp at E-P-sites and Arg-X-Lys at E-P-A-sites in HEK293 (Han et al., 2020) and poly-Lys or poly-Arg codons in yeast, scaling with the number of consecutive codons (Meydan and Guydosh, 2020). In yeast, synonymous codon composition of poly-Lys regions showed little effect on disome peaks, whereas poly-Arg disome peaks were increased by usage of CGA codons, which have been described to influence translation efficiency (Meydan and Guydosh, 2020). Dipeptide motifs previously described to slow translation due to interaction of adjacent tRNAs (Gamble et al., 2016) were strongly enriched in yeast disome profiling (Meydan and Guydosh, 2020). In HEK293 no clear evidence was found for increased collision due to high hydrophobicity of the nascent chain held in the ribosome exit tunnel (Han et al., 2020). Intriguingly, positive charging of the nascent chain only resulted in increased disome formation when a Pro-Pro motif was present in the E-P-site at the same time (Han et al., 2020). Moreover, neither tRNA abundance nor codon usage correlated with disome formation (Han et al., 2020). It is noteworthy that monosome profiling alone underestimated collision or pause sites in all mentioned studies, highlighting the benefit of profiling higher-order ribosome species.

These results, in part contradictory to each other, highlight the apparent underlying complexity of translation kinetics and ribosome pausing. Multiple approaches sought to pinpoint the influence of specific factors: with secondary structure predictions, Arpat *et al.* (2020) found that disome formation is enriched in unstructured regions directly downstream of a structured region (α-helix or β-sheet) (Arpat et al., 2020). In yeast, a fraction of collision sites were identified within domains, aligning with the observation of a previous study that ribosomes pause at the end of domains during recruitment of chaperones to facilitate peptide folding (Stein et al., 2019). Both findings, although conflicting at first glance, follow the hypothesis of programmed elongation slowdown to facilitate co-translational peptide folding or complex assembly. Another known programmed pause in translation occurs upon SRP binding to facilitate targeting to the ER, which was reflected in disome build-up in the signal peptide region with a steep decrease of disomes directly downstream (Arpat et al., 2020) (see discussion on Section 6.6). Analysis of evolutionary conservation indicates that disome sites are more conserved than expected by chance (Arpat et al., 2020). Disome sites are further associated with regulatory upstream ORFs, selenocysteine decoding, programmed ribosomal frameshifting, and co-translational processing of pro-ubiquitin and the XBP1 transcriptional factor (Arpat et al., 2020; Han et al., 2020; Meydan and Guydosh, 2020).

Whether or not all observed disomes represent terminally stalled translation events that need rescue and activation of the RQC remains elusive. Translational pauses, which may cause transient collisions, can have important physiological functions in promoting co-translational protein folding, protein targeting, and protein complex assembly (Collart and Weiss, 2020). Knockdown of ZNF598, the E3 ligase detecting terminally stalled ribosomes, did not lead to global changes in ribosome density at collision sites in HEK293 cells (Han et al., 2020). In yeast, depletion of the ZNF598 homolog Hel2 led to unexpected decrease in disome accumulation, for which the authors offer multiple explanations: ribosome collisions might happen frequently by chance, forming transient disomes, which, if not stabilized by Hel2, are resolved by resuming translation (Meydan and Guydosh, 2020). Hel2 deletion also resulted in the activation of the ISR, which attenuates protein synthesis through phosphorylation of the eIF2α translation initiation factor (Meydan and Guydosh, 2020). It is conceivable that ISR activation decreases ribosome loading onto mRNAs, limiting the chances of disome formation (Meydan and Guydosh, 2020). Indeed, it appears that not all endogenous disomes activate a quality control response. RNA sequencing revealed that the levels of most mRNAs were not changed by Hel2 deletion, indicating that disome formation, even when sensitive to the presence of Hel2, was not a strong trigger for mRNA degradation.

An additional layer of complexity was revealed by Stein *et al.* (2022), who studied the influence of ageing on ribosome pausing in yeast and nematode models (Stein et al., 2022). Although the cumulative pausing of ribosomes was not changed on a global scale, thousands of stall sites increased with age in both organisms (Stein et al., 2022). In yeast, pausing increased mostly on the amino acids Pro, Arg and Lys, independent of codon usage (Stein et al., 2022). Pausing on previously described sites, *i.e.* certain di- or tripeptides as well as polybasic motifs, was exacerbated upon ageing, in many cases leading to ribosomal collisions (Stein et al., 2022). Additionally, ageing impaired RQC processing of ribosome-stalled nascent chains, which can lead to their aggregation and is likely to contribute to age-dependent systemic decline (Stein et al., 2022).

## 8 Emerging co-translational quality control pathways

### 8.1 The UFMylation pathway: Eliminating ribosome-stalled nascent chains at the ER

Approximately one-third of the eukaryotic proteome is synthesized at the ER surface. Although the RQC pathway has been shown to also function at the ER (Crowder et al., 2015; von der Malsburg et al., 2015; Arakawa et al., 2016), recent studies indicate that cells employ additional mechanisms to clear nascent chains stalled at the Sec61 translocon. Wang et al. (2020) analyzed the fate of a non-stop model construct consisting of an ER-targeted GFP protein followed by a poly(A) sequence. The authors report that ribosomes stalled during the synthesis of this construct were conjugated with a Ubiquitin-fold modifier 1 (UFM1), which was required for the fast degradation of the translation-arrested protein product. UFMylation involves the sequential activation, conjugation, and ligation of UFM1 to a target substrate *via* an E1 (UBA5), E2 (UFC1), and E3 (UFL1) cascade that mirrors ubiquitin conjugation (Komatsu et al., 2004; Daniel and Liebau, 2014). Strikingly, ribosome UFMylation was not associated with nascent chain degradation by the proteasome. Instead, the UFM1 signal enabled the trafficking of the ER-localized stalled nascent chain to lysosomes for degradation (Stephani et al., 2020; Wang et al., 2020). The UFL1 E3 ligase forms a ternary complex with ER membrane adaptor DDRGK1 (also known as UFM1 binding protein-1, UFBP1) and CDK5RAP3 (also known as C53, LZAP, IC53, HSF-27) (Wu et al., 2010; Walczak et al., 2019; Stephani et al., 2020). CDK5RAP3 acts as a specificity determinant of the E3 ligase complex, restricting its activity towards the 40S ribosomal protein RPL26 (Peter et al., 2022). CDK5RAP3 can also act as an autophagy cargo receptor, as it interacts with ATG8, an ubiquitin-like protein that is conjugated to the phagophore upon activation of autophagy (Stephani et al., 2020). To date, the mechanisms recruiting the UFL1 ligase specifically to elongation-stalled ribosomes, or how the UFM1 modification in the ribosome promotes processing of the stalled nascent chain for degradation, remain to be defined. One important aspect to be addressed is the interplay between UFM1-dependent protein degradation and the RQC pathways: do they act on different sets of ER substrates, and do they share pathway components? It is also worth noting that the UFMylation system has pleiotropic roles in various cellular processes, including maintenance of ER homeostasis, autophagy, cell signaling, DNA damage response, transcriptional regulation, cell differentiation, and others [reviewed in (Fang and Pan, 2019; Gerakis et al., 2019; Banerjee et al., 2020; Witting and Mulder, 2021; Jing et al., 2022)]. It is not yet clear to what extent co-translational degradation contributes to the many phenotypes attributed to a dysfunction of the UFMylation system.

### 8.2 Protein quality control associated with nonsense-mediated mRNA decay

In addition to No-Go and Non-Stop decay, cells employ a third translation-dependent mRNA decay pathway in the cytosol. The function of the nonsense-mediated decay (NMD) is to degrade transcripts harboring a premature termination codon (PTC), which can arise from genetic mutations or stochastic errors in transcription or splicing (Chang et al., 1979; Losson and Lacroute, 1979; Maquat et al., 1981; Kinniburgh et al., 1982). In addition to this mRNA quality control function, NMD also targets a substantial part of the transcriptome as a form of post-transcriptional regulation (Lelivelt and Culbertson, 1999; He et al., 2003; Mendell et al., 2004; Rehwinkel et al., 2005). It has been suggested that ribosome stalling at a termination codon indicates aberrant translation termination and thereby triggers NMD (Amrani et al., 2004; Peixeiro et al., 2012), although a subsequent study indicated that ribosome occupancy at the termination codon is similar for NMD-sensitive and -insensitive transcripts (Karousis et al., 2020). NMD can be activated through multiple mechanisms. For example, when the terminating ribosome is located far away from the poly(A) sequence, it fails to interact with the poly(A)-binding protein (PABPC1), which promotes proper translation termination (Amrani et al., 2004). NMD can also be promoted by proximity of the terminating ribosome with an exon junction complex (EJC), which interacts with NMD factors (Kim et al., 2001; Le Hir et al., 2001). Similar to non-stop and no-go transcripts, PTC-containing messages produce defective, truncated proteins that can potentially harm the cell by misfolding and aggregating or by assuming dominant-negative effects. Indeed, proteins produced by NMD-targeted transcripts are markedly less stable than proteins produced by normal transcripts, even when the generated products are identical (Chu et al., 2021; Udy and Bradley, 2022). This observation strongly corroborates that premature termination activates a quality control pathway to degrade the resulting polypeptide. Protein degradation is reduced upon depletion of central NMD factors UPF1 or SMG1, indicating that they participate in the activation of protein quality control (Chu et al., 2021). As NMD factors are recruited to PTC-located ribosomes, it is very likely that the associated quality control pathway acts co-translationally.

**FIGURE 8:**
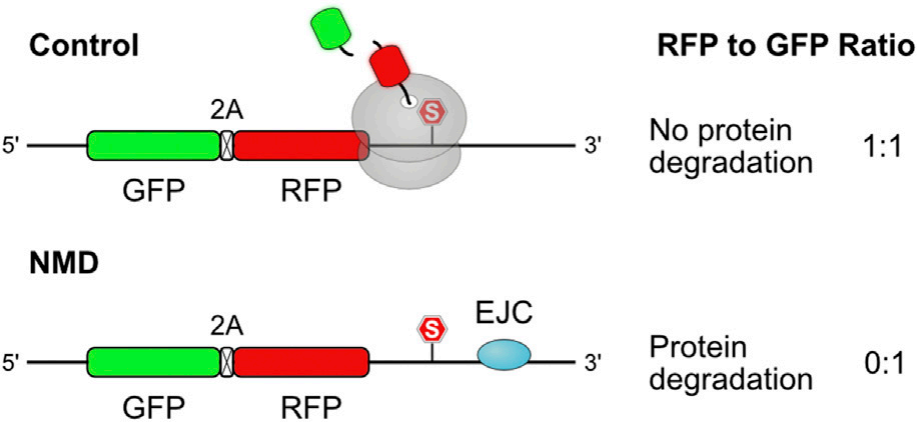
Dual fluorescence reporter assay for measuring NMD-associated protein degradation. Two fluorescent proteins, separated by a 2A peptide bond skipping sequence, are produced in equal amounts from the same mRNA. Introduction of an intron after the stop (S) codon results in deposition of an exon junction complex (EJC) in the 3′ UTR during splicing. The EJC downstream of the termination codon is perceived as a signal for NMD activation. When ribosomes reach the termination codon, the GFP protein has already been released, while RFP remains associated to the ribosome until termination is completed. NMD-induced co-translational degradation of the RFP protein results in decreased RFP to GFP ratios of the EJC-containing construct in flow cytometry analysis. As GFP serves as a normalizing factor for expression, changes in mRNA levels due to mRNA decay do not influence the protein degradation readout.

Since NMD decay involves mRNA cleavage in close proximity to the PTC (Huntzinger et al., 2008; Eberle et al., 2009), it is conceivable that it may elicit ribosomal stalling at the neo 3′ end, the signal for NSD mRNA decay and RQC-mediated protein degradation. In accordance, a study in *Caenorhabditis elegans* has shown that deletion of NSD factors pelo-1/skih-2 (homologs of Pelota and the RNA helicase Ski2, involved in mRNA decay) leads to the accumulation of ribosomes at the 3′ end of mRNAs truncated near the stop codon, in endogenous NMD-regulated transcripts (Arribere and Fire, 2018). Whether these NMD-generated non-stop transcripts also activate the RQC remains to be formally addressed. The RQC (Ltn1 and Rqc1) was found to be involved in the degradation of Orf1p, a truncated peptide originated from a naturally occurring PTC in the ornithine decarboxylase antizyme 1 (*OAZ1*) mRNA in yeast (Pradhan et al., 2021). However, the RQC might not be the primary degradation pathway for all classes of NMD targets. In yeast, the stability of endogenous phosphogluconate dehydrogenase truncated by a premature termination codon (Gnd1^PTC^) was increased by deletion of the E3 ubiquitin ligases Upf1, a component of the NMD pathway, and the N-end rule ligase Ubr1, which is also involved in the degradation of misfolded cytosolic proteins (Eisele and Wolf, 2008; Heck et al., 2010; Nillegoda et al., 2010), while deletion of the RQC ubiquitin ligase Ltn1 had no effect (Verma et al., 2013). Chu *et al.* analyzed the stability of a dual fluorescence reporter containing an EJC in the 3′ UTR of the corresponding transcript in HEK293 human cells (Figure 8). Although this reporter was stabilized by depletion of NMD factors and by proteasomal inhibition, it was not affected by knockdown of the core RQC components Listerin and NEMF (Chu et al., 2021). A study in pre-print using a similar NMD reporter protein confirmed that the RQC did not participate in its degradation (Inglis et al., 2022). Using genetic screens, the authors identified the E3 ubiquitin ligase CNOT4 to be involved in NMD-dependent degradation of this reporter. As the employed dual fluorescence assay is not influenced by mRNA stability, the authors propose that the quality control role of CNOT4 may be independent of its function in the multi-subunit CCR4-NOT complex, which regulates eukaryotic gene expression by deadenylation (Inglis et al., 2022). Interestingly, CNOT4 knockdown was previously shown to reduce total co-translational ubiquitination levels in 10% in human 293T cells (Wang et al., 2013), while in yeast NOT4 deletion largely increased co-translational ubiquitination, likely reflecting the influence of CCR4-NOT complex in mRNA quality control (Duttler et al., 2013).

In conclusion, a growing body of work has indicated that NMD activates co-translational degradation of polypeptides produced by PTC-containing transcripts. The protein quality control factors involved in this process are only starting to emerge, and might differ between classes of NMD substrates. It will be interesting to see if the factors engaged in a quality control function, i.e., in eliminating defective PTC-truncated proteins, might also assume a role in silencing of NMD-regulated transcripts. Given that NMD alters the expression of ∼10% of transcripts in a wide variety of eukaryotes (Huang and Wilkinson, 2012), and that it plays an essential role in organism development, cell differentiation, and disease physiology, elucidating the associated protein quality control mechanisms represents a topic of high interest.

### 8.3 Quality control associated with dysfunctional ribosomes

In budding yeast, ribosomal RNAs containing point mutations that adversely affect ribosome decoding function lead to 18S rRNA clearance by a quality control mechanism known as non-functional 18S rRNA decay (NRD) (LaRiviere et al., 2006). Activation of NRD involves polyubiquitination of the 40S protein RPS3 and ribosomal splitting, through a multi-tiered process consisting of mono-ubiquitination and ubiquitin chain elongation by the Mag2 and Fap1 ubiquitin ligases, respectively (Sugiyama et al., 2019). Interestingly, while the NRD-inducing 18S base mutation A1755C led to 80S ribosomes arrested at the initiation codon, selective ribosome profiling experiments showed that both Mag2 and Fap1 prominently associate with ribosomes located within the coding sequence (Li et al., 2022). This observation indicates that NRD decay also targets translating ribosomes, suggesting that defective, truncated nascent proteins might also be produced in the context of NRD-eliciting situations. It remains to be defined whether NRD is associated with co-translational quality control pathway(s) for nascent chain degradation.

Importantly, the mechanism of NRD activation seems to be quite distinct from sensing of defective mRNAs by the NSD/NGD and RQC pathways. Fap1, contrary to ZNF598, shows a strong preference for binding to monosomes over polysomes (Li et al., 2022). Cryo-EM analysis revealed that the elongated Fap1 structure envelops the 40S subunit, simultaneously binding to mRNA both at the entry and exit sites of the ribosome. The authors hypothesize that movement of mRNA through the ribosome could destabilize these Fap1 contact sites, and therefore Fap1 would only be able to stably interact with and modify stalled 80S monosomes (Li et al., 2022). The positioning of Fap1 sterically clashes with the canonical structure of collided ribosomes, indicating that i) Fap1 biding would hinder the formation of disomes, and ii) Fap1 would not be able to bind to pre-formed disomes. The apparent preference for monosomes raises interesting questions about the physiological function of NRD, as normally only few transcripts are translated as monosomes.

The human homolog of Mag2, RNF10, has been implicated in the selective degradation of 40S (but not 60S) ribosomal proteins (Garshott et al., 2021). A number of pharmacological treatments have been shown to induce RNF10-mediated ubiquitination of 40S proteins RPS3 and RPS2 in human cells: harringtonine and lactimidomycin, which inhibit progression of 80S ribosomes at the start codon, rocaglates and pateamine A, which impair mRNA scanning by the pre-initiation complex, and cycloheximide in high concentration, which globally inhibits ribosome elongation. The authors propose that impeding progression of scanning or elongating ribosomes near start codons induces site-specific ribosome ubiquitination and 40S protein degradation by a pathway they named initiation RQC (iRQC) (Garshott et al., 2021). Further studies will be necessary to define how often and how far iRQC-targeted small ribosomal subunits are able to progress into translation, at risk of producing defective protein products.

In conclusion, a growing body of work revealed that cells survey and respond to dysfunctional ribosomes with rRNA and ribosomal protein degradation. Although the mechanisms mediating these responses are only starting to emerge, current evidence indicates that ribosomes engaged in translation are also targeted for degradation. The exact physiological triggers of these responses, and how cells cope with nascent polypeptides produced by these defective ribosomes, represent important points of future investigation.

## 9 Quality control pathways recruited to active translation complexes

While there is considerable evidence that nascent proteins can become ubiquitinated while associated with elongating ribosomes (see Section 3), the mechanisms and the functional relevance of these processes remain elusive. Duttler *et al.* analyzed the total co-translational ubiquitination levels in a panel of 10 yeast single and double deletion strains of ubiquitin-conjugating (E2) and ubiquitin ligase (E3) enzymes. Double deletion of the E2 enzymes UBC1/4, but not single deletions, led to a strong (∼50%) reduction in co-translational ubiquitination. Deleting single E3 ligases led to no or very small (5%–10%) effects. The latter was the case for proteasome-linked Hul5, and the major ER-associated protein degradation (ERAD) components HRD1 and DOA10 (Duttler et al., 2013). In human cells, knockout of chaperone-associated E3 ligase CHIP, the Hul5 homolog UBE3C, or double deletion of N-end rule pathway E3 ligases UBR1/2 did not affect total levels of co-translational ubiquitination (Wang et al., 2013). However, in yeast the extent of co-translational degradation of a β-galactosidase derived protein carrying a strong N-terminal degradation signal was influenced by the levels of the Ubr1 E3 ligase (Turner and Varshavsky, 2000). Although these experiments did not differentiate between stalled and active translation, the potential implication of general quality control factors in co-translational degradation indicates that some ubiquitin ligases are able to function both *on* and *off* the ribosome. The involvement of general quality control players is also in line with observations that CTU^A^ events are promoted by protein misfolding (Wang et al., 2013).

Tian *et al.* (2021) showed that HSP70 chaperone HSPA1 supports the co-translational degradation of nascent polypeptides during proteotoxic stress. In cultured mouse cells, HSPA1 was upregulated and remained elevated following transient heat shock, contributing to the development of thermotolerance. Sucrose fractionation experiments showed that heat shock led to the accumulation of K48-polyubiquitinated proteins in polysomal fractions. The majority of these ubiquitinated proteins corresponded to nascent chains rather than ribosomal proteins, as they were displaced from polysomes by puromycin treatment. Interestingly, pre-exposed thermotolerant cells did not show an accumulation of ribosome-associated polyubiquitinated proteins following a second heat exposure. However, treatment of these cells with Hsp70-or proteasomal-inhibitors restored the buildup of K48-modified nascent chains. Hsp70 activity also increased the recruitment of proteasomes to polysomes upon stress. While the majority of polyubiquitinated proteins formed upon proteasomal inhibition remained soluble, Hsp70 inhibition led to their accumulation in the insoluble fraction. The authors conclude that Hsp70 promotes proteasomal degradation of nascent and newly synthesized K48-polyubiquitylated proteins during heat stress, most likely by keeping them in a soluble state. In accordance, deletion of the HSPA1 co-chaperone HSPH1 impeded thermotolerance and tumor growth in mice (Tian et al., 2021). While it is not yet clear if heat-induced nascent chain ubiquitination happens in elongating or stalled ribosomes, the study highlights the importance of eliminating damaged nascent proteins for cell survival during stress.

Overall, the limited amount of evidence available at present indicates that co-translational ubiquitination of actively translation complexes might be carried out by multiple ubiquitin ligases, following nascent chain conformational cues, exposed degron sequences, and/or chaperone binding. Nevertheless, it is difficult to reconcile how recognition of abnormal co-translational folding would work, since, apart from a few exceptions, proteins in the process of being synthesized are normally non-native (Rodrigo-Brenni and Hegde, 2012). In general terms, the vicinity of the ribosome represents an environment that disfavors ubiquitination—not only due to the shielding effect of the ribosome itself, but also by the action of ribosome-bound molecular chaperones and by the occurrence of co-translational folding events (Duttler et al., 2013). Moreover, ubiquitin modifications can be removed by the action of deubiquitinating enzymes, meaning that not all co-translationally ubiquitination events may lead to nascent chain degradation. On the other hand, co-translational triaging of folding-incompetent nascent proteins can have important advantages towards minimizing the risk of proteostasis imbalance, by limiting the burden on the proteostasis network, and by potentially creating redundancy with downstream quality control pathways (Rodrigo-Brenni and Hegde, 2012). It has also been proposed that ubiquitination of nascent proteins may serve as a “fix me” signal with multiple possible purposes, such as shielding hydrophobic sequences, preventing enzymatic activity or inappropriate interactions during trafficking, and recruiting ubiquitin-dependent chaperones and unfolding enzymes (Culver et al., 2022). All these possibilities underscore the complexity of balancing protein synthesis efficiency with the risk of proteostasis failure, and the need to address the underlying mechanisms in the future.

## 10 Future perspectives

Research in the past decade revealed that the ribosome–or more specifically the speed of the ribosome–plays a seminal role in activating co-translational protein and mRNA quality control pathways that are essential for organismal fitness. While it is clear that the RQC pathway acts to prevent the detrimental effects arising from errors in mRNA synthesis and/or environmental mRNA damage, whether it also responds to cleaved mRNA byproducts resulting from physiological processes, such as RIDD, NMD, mRNA silencing, etc, remains to be addressed. Moreover, new evidence indicates that defective mRNAs might not be the only cause of RQC activation; failure in co-translational processes, such as transmembrane protein insertion, seems to activate protein degradation as well. Further studies will be necessary to understand i) how co-translational processes influence ribosomal elongation, and ii) whether the associated quality control pathway(s) differ in any way from the canonical RQC. Clarifying whether cells distinguish between these essentially different translation defects will be specially relevant considering the emerging role of ribosome collisions as a signal of stress, controlling important cell fate decisions such as the integrated stress response, cell cycle progression, the ribotoxic stress response (apoptosis), and interferon gene expression (Wu et al., 2020; Wan et al., 2021; Yan and Zaher, 2021; Kim and Zaher, 2022; Robinson et al., 2022; Stoneley et al., 2022). The interplay between the RQC and novel pathways responding to specific types of mRNA defects/ribosome stalls also represents an important aspect of future investigation. It remains to be defined whether ribosome-restricted quality control pathways exist outside the context of stalled elongation. It has been shown that some ubiquitin ligases are able to ubiquitinate substrates both at the ribosome and in the cytosol, but whether such co-translational ubiquitination events contribute to proteostasis balance or represent off target reactions warrants further investigation. New technologies, such as ribosome profiling and dual fluorescence reporters coupled to genetic screens, together with *in vitro* reconstitution and structural analyses, will help to delineate these open questions.

## References

[B1] Adamian L. Liang J. (2002). Interhelical hydrogen bonds and spatial motifs in membrane proteins: Polar clamps and serine zippers. Proteins 47 (2), 209–218. 10.1002/prot.10071 11933067

[B2] Alhebshi A. Sideri T. C. Holland S. L. Avery S. V. (2012). The essential iron-sulfur protein Rli1 is an important target accounting for inhibition of cell growth by reactive oxygen species. Mol. Biol. Cell 23 (18), 3582–3590. 10.1091/mbc.E12-05-0413 22855532 PMC3442406

[B3] Amrani N. Ganesan R. Kervestin S. Mangus D. A. Ghosh S. Jacobson A. (2004). A faux 3'-UTR promotes aberrant termination and triggers nonsense-mediated mRNA decay. Nature 432 (7013), 112–118. 10.1038/nature03060 15525991

[B4] Annibaldis G. Domanski M. Dreos R. Contu L. Carl S. Klay N. (2020). Readthrough of stop codons under limiting ABCE1 concentration involves frameshifting and inhibits nonsense-mediated mRNA decay. Nucleic Acids Res. 48 (18), 10259–10279. 10.1093/nar/gkaa758 32941650 PMC7544199

[B5] Arakawa S. Yunoki K. Izawa T. Tamura Y. Nishikawa S. Endo T. (2016). Quality control of nonstop membrane proteins at the ER membrane and in the cytosol. Sci. Rep. 6, 30795. 10.1038/srep30795 27481473 PMC4969602

[B6] Arpat A. B. Liechti A. De Matos M. Dreos R. Janich P. Gatfield D. (2020). Transcriptome-wide sites of collided ribosomes reveal principles of translational pausing. Genome Res. 30 (7), 985–999. 10.1101/gr.257741.119 32703885 PMC7397865

[B7] Arribere J. A. Fire A. Z. (2018). Nonsense mRNA suppression via nonstop decay. Elife 7, 332922–e33323. 10.7554/eLife.33292 PMC5777819 29309033

[B8] Arthur L. Pavlovic-Djuranovic S. Smith-Koutmou K. Green R. Szczesny P. Djuranovic S. (2015). Translational control by lysine-encoding A-rich sequences. Sci. Adv. 1 (6), e1500154. 10.1126/sciadv.1500154 26322332 PMC4552401

[B9] Banerjee S. Kumar M. Wiener R. (2020). Decrypting UFMylation: How proteins are modified with UFM1. Biomolecules 10 (10), 1442–1514. 10.3390/biom10101442 33066455 PMC7602216

[B10] Bano-Polo M. Baeza-Delgado C. Tamborero S. Hazel A. Grau B. Nilsson I. (2018). Transmembrane but not soluble helices fold inside the ribosome tunnel. Nat. Commun. 9 (1), 5246. 10.1038/s41467-018-07554-7 30531789 PMC6286305

[B11] Barthelme D. Scheele U. Dinkelaker S. Janoschka A. Macmillan F. Albers S. V. (2007). Structural organization of essential iron-sulfur clusters in the evolutionarily highly conserved ATP-binding cassette protein ABCE1. J. Biol. Chem. 282 (19), 14598–14607. 10.1074/jbc.M700825200 17355973

[B12] Beck M. Schmidt A. Malmstroem J. Claassen M. Ori A. Szymborska A. (2011). The quantitative proteome of a human cell line. Mol. Syst. Biol. 7 (1), 549. 10.1038/msb.2011.82 22068332 PMC3261713

[B13] Bengtson M. H. Joazeiro C. A. (2010). Role of a ribosome-associated E3 ubiquitin ligase in protein quality control. Nature 467 (7314), 470–473. 10.1038/nature09371 20835226 PMC2988496

[B14] Blobel G. (1980). Intracellular protein topogenesis. Proc. Natl. Acad. Sci. U. S. A. 77 (3), 1496–1500. 10.1073/pnas.77.3.1496 6929499 PMC348522

[B15] Brandman O. Hegde R. S. (2016). Ribosome-associated protein quality control. Nat. Struct. Mol. Biol. 23 (1), 7–15. 10.1038/nsmb.3147 26733220 PMC4853245

[B16] Brandman O. Stewart-Ornstein J. Wong D. Larson A. Williams C. C. Li G. W. (2012). A ribosome-bound quality control complex triggers degradation of nascent peptides and signals translation stress. Cell 151 (5), 1042–1054. 10.1016/j.cell.2012.10.044 23178123 PMC3534965

[B17] Chandrasekaran V. Juszkiewicz S. Choi J. Puglisi J. D. Brown A. Shao S. (2019). Mechanism of ribosome stalling during translation of a poly(A) tail. Nat. Struct. Mol. Biol. 26 (12), 1132–1140. 10.1038/s41594-019-0331-x 31768042 PMC6900289

[B18] Chang J. C. Temple G. F. Trecartin R. F. Kan Y. W. (1979). Suppression of the nonsense mutation in homozygous beta 0 thalassaemia. Nature 281 (5732), 602–603. 10.1038/281602a0 492326

[B19] Chitwood P. J. Juszkiewicz S. Guna A. Shao S. Hegde R. S. (2018). EMC is required to initiate accurate membrane protein topogenesis. Cell 175 (6), 1507–1519. e1516. 10.1016/j.cell.2018.10.009 30415835 PMC6269167

[B20] Choe Y. J. Park S. H. Hassemer T. Korner R. Vincenz-Donnelly L. Hayer-Hartl M. (2016). Failure of RQC machinery causes protein aggregation and proteotoxic stress. Nature 531 (7593), 191–195. 10.1038/nature16973 26934223

[B21] Chu J. Hong N. A. Masuda C. A. Jenkins B. V. Nelms K. A. Goodnow C. C. (2009). A mouse forward genetics screen identifies LISTERIN as an E3 ubiquitin ligase involved in neurodegeneration. Proc. Natl. Acad. Sci. U. S. A. 106 (7), 2097–2103. 10.1073/pnas.0812819106 19196968 PMC2650114

[B22] Chu V. Feng Q. Lim Y. Shao S. (2021). Selective destabilization of polypeptides synthesized from NMD-targeted transcripts. Mol. Biol. Cell 32 (22), ar38. 10.1091/mbc.E21-08-0382 34586879 PMC8694075

[B23] Collart M. A. Weiss B. (2020). Ribosome pausing, a dangerous necessity for co-translational events. Nucleic Acids Res. 48 (3), 1043–1055. 10.1093/nar/gkz763 31598688 PMC7026645

[B24] Costa E. A. Subramanian K. Nunnari J. Weissman J. S. (2018). Defining the physiological role of SRP in protein-targeting efficiency and specificity. Science 359 (6376), 689–692. 10.1126/science.aar3607 29348368 PMC5970945

[B25] Crowder J. J. Geigges M. Gibson R. T. Fults E. S. Buchanan B. W. Sachs N. (2015). Rkr1/Ltn1 ubiquitin ligase-mediated degradation of translationally stalled endoplasmic reticulum proteins. J. Biol. Chem. 290 (30), 18454–18466. 10.1074/jbc.M115.663559 26055716 PMC4513105

[B26] Culver J. A. Li X. Jordan M. Mariappan M. (2022). A second chance for protein targeting/folding: Ubiquitination and deubiquitination of nascent proteins. Bioessays 44 (6), e2200014. 10.1002/bies.202200014 35357021 PMC9133216

[B27] Cymer F. Ismail N. von Heijne G. (2014). Weak pulling forces exerted on Nin-orientated transmembrane segments during co-translational insertion into the inner membrane of *Escherichia coli* . FEBS Lett. 588 (10), 1930–1934. 10.1016/j.febslet.2014.03.050 24726730

[B28] Cymer F. von Heijne G. (2013). Cotranslational folding of membrane proteins probed by arrest-peptide-mediated force measurements. Proc. Natl. Acad. Sci. U. S. A. 110 (36), 14640–14645. 10.1073/pnas.1306787110 23959879 PMC3767533

[B29] D'Orazio K. N. Wu C. C. Sinha N. Loll-Krippleber R. Brown G. W. Green R. (2019). The endonuclease Cue2 cleaves mRNAs at stalled ribosomes during No Go Decay. Elife 8, e49117–e49127. 10.7554/eLife.49117 31219035 PMC6598757

[B30] Daniel J. Liebau E. (2014). The ufm1 cascade. Cells 3 (2), 627–638. 10.3390/cells3020627 24921187 PMC4092871

[B31] Davies M. V. Chang H. W. Jacobs B. L. Kaufman R. J. (1993). The E3L and K3L vaccinia virus gene products stimulate translation through inhibition of the double-stranded RNA-dependent protein kinase by different mechanisms. J. Virol. 67 (3), 1688–1692. 10.1128/JVI.67.3.1688-1692.1993 8094759 PMC237544

[B32] Davies M. V. Elroy-Stein O. Jagus R. Moss B. Kaufman R. J. (1992). The vaccinia virus K3L gene product potentiates translation by inhibiting double-stranded-RNA-activated protein kinase and phosphorylation of the alpha subunit of eukaryotic initiation factor 2. J. Virol. 66 (4), 1943–1950. 10.1128/JVI.66.4.1943-1950.1992 1347793 PMC288982

[B33] Defenouillere Q. Namane A. Mouaikel J. Jacquier A. Fromont-Racine M. (2017). The ribosome-bound quality control complex remains associated to aberrant peptides during their proteasomal targeting and interacts with Tom1 to limit protein aggregation. Mol. Biol. Cell 28 (9), 1165–1176. 10.1091/mbc.E16-10-0746 28298488 PMC5415013

[B34] Defenouillere Q. Yao Y. Mouaikel J. Namane A. Galopier A. Decourty L. (2013). Cdc48-associated complex bound to 60S particles is required for the clearance of aberrant translation products. Proc. Natl. Acad. Sci. U. S. A. 110 (13), 5046–5051. 10.1073/pnas.1221724110 23479637 PMC3612664

[B35] Defenouillere Q. Zhang E. Namane A. Mouaikel J. Jacquier A. Fromont-Racine M. (2016). Rqc1 and Ltn1 prevent C-terminal alanine-threonine tail (CAT-tail)-induced protein aggregation by efficient recruitment of Cdc48 on stalled 60S subunits. J. Biol. Chem. 291 (23), 12245–12253. 10.1074/jbc.M116.722264 27129255 PMC4933273

[B36] den Brave F. Engelke J. Becker T. (2021). Quality control of protein import into mitochondria. Biochem. J. 478 (16), 3125–3143. 10.1042/BCJ20190584 34436539

[B37] DiGiuseppe S. Rollins M. G. Bartom E. T. Walsh D. (2018). ZNF598 plays distinct roles in interferon-stimulated gene expression and poxvirus protein synthesis. Cell Rep. 23 (5), 1249–1258. 10.1016/j.celrep.2018.03.132 29719242 PMC5951170

[B38] Doma M. K. Parker R. (2006). Endonucleolytic cleavage of eukaryotic mRNAs with stalls in translation elongation. Nature 440 (7083), 561–564. 10.1038/nature04530 16554824 PMC1839849

[B39] dos Reis. M. Savva R. Wernisch L. (2004). Solving the riddle of codon usage preferences: A test for translational selection. Nucleic Acids Res. 32 (17), 5036–5044. 10.1093/nar/gkh834 15448185 PMC521650

[B40] Duttler S. Pechmann S. Frydman J. (2013). Principles of cotranslational ubiquitination and quality control at the ribosome. Mol. Cell 50 (3), 379–393. 10.1016/j.molcel.2013.03.010 23583075 PMC3886275

[B41] Eberle A. B. Lykke-Andersen S. Muhlemann O. Jensen T. H. (2009). SMG6 promotes endonucleolytic cleavage of nonsense mRNA in human cells. Nat. Struct. Mol. Biol. 16 (1), 49–55. 10.1038/nsmb.1530 19060897

[B42] Eisele F. Wolf D. H. (2008). Degradation of misfolded protein in the cytoplasm is mediated by the ubiquitin ligase Ubr1. FEBS Lett. 582 (30), 4143–4146. 10.1016/j.febslet.2008.11.015 19041308

[B43] Fang Z. Pan Z. (2019). Essential role of Ubiquitin-fold modifier 1 conjugation in DNA damage response. DNA Cell Biol. 38 (10), 1030–1039. 10.1089/dna.2019.4861 31368785

[B44] Frischmeyer P. A. van Hoof A. O'Donnell K. Guerrerio A. L. Parker R. Dietz H. C. (2002). An mRNA surveillance mechanism that eliminates transcripts lacking termination codons. Science 295 (5563), 2258–2261. 10.1126/science.1067338 11910109

[B45] Gamble C. E. Brule C. E. Dean K. M. Fields S. Grayhack E. J. (2016). Adjacent codons act in concert to modulate translation efficiency in yeast. Cell 166 (3), 679–690. 10.1016/j.cell.2016.05.070 27374328 PMC4967012

[B46] Garshott D. M. An H. Sundaramoorthy E. Leonard M. Vicary A. Harper J. W. (2021). iRQC, a surveillance pathway for 40S ribosomal quality control during mRNA translation initiation. Cell Rep. 36 (9), 109642. 10.1016/j.celrep.2021.109642 34469731 PMC8997904

[B47] Gerakis Y. Quintero M. Li H. Hetz C. (2019). The UFMylation system in proteostasis and beyond. Trends Cell Biol. 29 (12), 974–986. 10.1016/j.tcb.2019.09.005 31703843 PMC6917045

[B48] Glover M. L. Burroughs A. M. Monem P. C. Egelhofer T. A. Pule M. N. Aravind L. (2020). NONU-1 encodes a conserved endonuclease required for mRNA translation surveillance. Cell Rep. 30 (13), 4321–4331. e4324. 10.1016/j.celrep.2020.03.023 32234470 PMC7184879

[B49] Goldman D. H. Livingston N. M. Movsik J. Wu B. Green R. (2021). Live-cell imaging reveals kinetic determinants of quality control triggered by ribosome stalling. Mol. Cell 81 (8), 1830–1840.e8. 10.1016/j.molcel.2021.01.029 33581075 PMC8052310

[B50] Guna A. Volkmar N. Christianson J. C. Hegde R. S. (2018). The ER membrane protein complex is a transmembrane domain insertase. Science 359 (6374), 470–473. 10.1126/science.aao3099 29242231 PMC5788257

[B51] Guydosh N. R. Green R. (2014). Dom34 rescues ribosomes in 3' untranslated regions. Cell 156 (5), 950–962. 10.1016/j.cell.2014.02.006 24581494 PMC4022138

[B52] Han P. Shichino Y. Schneider-Poetsch T. Mito M. Hashimoto S. Udagawa T. (2020). Genome-wide survey of ribosome collision. Cell Rep. 31 (5), 107610. 10.1016/j.celrep.2020.107610 32375038 PMC7746506

[B53] He F. Li X. Spatrick P. Casillo R. Dong S. Jacobson A. (2003). Genome-wide analysis of mRNAs regulated by the nonsense-mediated and 5' to 3' mRNA decay pathways in yeast. Mol. Cell 12 (6), 1439–1452. 10.1016/s1097-2765(03)00446-5 14690598

[B54] Heck J. W. Cheung S. K. Hampton R. Y. (2010). Cytoplasmic protein quality control degradation mediated by parallel actions of the E3 ubiquitin ligases Ubr1 and San1. Proc. Natl. Acad. Sci. U. S. A. 107 (3), 1106–1111. 10.1073/pnas.0910591107 20080635 PMC2824284

[B55] Hickey K. L. Dickson K. Cogan J. Z. Replogle J. M. Schoof M. D'Orazio K. N. (2020). GIGYF2 and 4EHP inhibit translation initiation of defective messenger RNAs to assist ribosome-associated quality control. Mol. Cell 79 (6), 950–962. 10.1016/j.molcel.2020.07.007 32726578 PMC7891188

[B56] Hilal T. Yamamoto H. Loerke J. Burger J. Mielke T. Spahn C. M. (2016). Structural insights into ribosomal rescue by Dom34 and Hbs1 at near-atomic resolution. Nat. Commun. 7 (1), 13521. 10.1038/ncomms13521 27995908 PMC5187420

[B57] Hipp M. S. Park S. H. Hartl F. U. (2014). Proteostasis impairment in protein-misfolding and -aggregation diseases. Trends Cell Biol. 24 (9), 506–514. 10.1016/j.tcb.2014.05.003 24946960

[B58] Hollien J. Lin J. H. Li H. Stevens N. Walter P. Weissman J. S. (2009). Regulated Ire1-dependent decay of messenger RNAs in mammalian cells. J. Cell Biol. 186 (3), 323–331. 10.1083/jcb.200903014 19651891 PMC2728407

[B59] Hollien J. Weissman J. S. (2006). Decay of endoplasmic reticulum-localized mRNAs during the unfolded protein response. Science 313 (5783), 104–107. 10.1126/science.1129631 16825573

[B60] Huang L. Wilkinson M. F. (2012). Regulation of nonsense-mediated mRNA decay. Wiley Interdiscip. Rev. RNA 3 (6), 807–828. 10.1002/wrna.1137 23027648

[B61] Huntzinger E. Kashima I. Fauser M. Sauliere J. Izaurralde E. (2008). SMG6 is the catalytic endonuclease that cleaves mRNAs containing nonsense codons in metazoan. RNA 14 (12), 2609–2617. 10.1261/rna.1386208 18974281 PMC2590965

[B62] Ikeuchi K. Tesina P. Matsuo Y. Sugiyama T. Cheng J. Saeki Y. (2019). Collided ribosomes form a unique structural interface to induce Hel2-driven quality control pathways. EMBO J. 38 (5), e100276. 10.15252/embj.2018100276 30609991 PMC6396155

[B63] Inglis A. J. Guna A. Merchán Á. G. Pal A. Esantsi T. K. Keys H. R. (2022). Coupled protein quality control during nonsense mediated mRNA decay. bioRxiv 2021, 473893. 10.1101/2021.12.22.473893 PMC10234110 37218462

[B64] Ingolia N. T. Ghaemmaghami S. Newman J. R. Weissman J. S. (2009). Genome-wide analysis *in vivo* of translation with nucleotide resolution using ribosome profiling. Science 324 (5924), 218–223. 10.1126/science.1168978 19213877 PMC2746483

[B65] Ingolia N. T. Lareau L. F. Weissman J. S. (2011). Ribosome profiling of mouse embryonic stem cells reveals the complexity and dynamics of mammalian proteomes. Cell 147 (4), 789–802. 10.1016/j.cell.2011.10.002 22056041 PMC3225288

[B66] Jing Y. Mao Z. Chen F. (2022). UFMylation system: An emerging player in tumorigenesis. Cancers (Basel) 14 (14), 3501. 10.3390/cancers14143501 35884562 PMC9323365

[B67] Joazeiro C. A. P. (2017). Ribosomal stalling during translation: Providing substrates for ribosome-associated protein quality control. Annu. Rev. Cell Dev. Biol. 33 (1), 343–368. 10.1146/annurev-cellbio-111315-125249 28715909

[B68] Jomaa A. Gamerdinger M. Hsieh H.-H. Wallisch A. Chandrasekaran V. Ulusoy Z. (2022). Mechanism of signal sequence handover from NAC to SRP on ribosomes during ER-protein targeting. Science 375 (6583), 839–844. 10.1126/science.abl6459 35201867 PMC7612438

[B69] Juszkiewicz S. Chandrasekaran V. Lin Z. Kraatz S. Ramakrishnan V. Hegde R. S. (2018). ZNF598 is a quality control sensor of collided ribosomes. Mol. Cell 72 (3), 469–481. 10.1016/j.molcel.2018.08.037 30293783 PMC6224477

[B70] Juszkiewicz S. Hegde R. S. (2017). Initiation of quality control during poly(A) translation requires site-specific ribosome ubiquitination. Mol. Cell 65 (4), 743–750. 10.1016/j.molcel.2016.11.039 28065601 PMC5316413

[B71] Kalthoff K. Urban K. Jackle H. (1978). Photoreactivation of RNA in UV-irradiated insect eggs (Smittia sp., Chironomidae, Diptera) II. Evidence for heterogeneous light-dependent repair activities. Photochem Photobiol. 27 (3), 317–322. 10.1111/j.1751-1097.1978.tb07606.x 569866

[B72] Karousis E. D. Gurzeler L. A. Annibaldis G. Dreos R. Muhlemann O. (2020). Human NMD ensues independently of stable ribosome stalling. Nat. Commun. 11 (1), 4134. 10.1038/s41467-020-17974-z 32807779 PMC7431590

[B73] Kim K. Q. Zaher H. S. (2022). Canary in a coal mine: Collided ribosomes as sensors of cellular conditions. Trends Biochem. Sci. 47 (1), 82–97. 10.1016/j.tibs.2021.09.001 34607755 PMC8688274

[B74] Kim V. N. Kataoka N. Dreyfuss G. (2001). Role of the nonsense-mediated decay factor hUpf3 in the splicing-dependent exon-exon junction complex. Science 293 (5536), 1832–1836. 10.1126/science.1062829 11546873

[B75] Kinniburgh A. J. Maquat L. E. Schedl T. Rachmilewitz E. Ross J. (1982). mRNA-deficient beta o-thalassemia results from a single nucleotide deletion. Nucleic Acids Res. 10 (18), 5421–5427. 10.1093/nar/10.18.5421 6292840 PMC320886

[B76] Kirchner S. Cai Z. Rauscher R. Kastelic N. Anding M. Czech A. (2017). Alteration of protein function by a silent polymorphism linked to tRNA abundance. PLoS Biol. 15 (5), e2000779. 10.1371/journal.pbio.2000779 28510592 PMC5433685

[B77] Komatsu M. Chiba T. Tatsumi K. Iemura S. Tanida I. Okazaki N. (2004). A novel protein-conjugating system for Ufm1, a ubiquitin-fold modifier. EMBO J. 23 (9), 1977–1986. 10.1038/sj.emboj.7600205 15071506 PMC404325

[B78] Kostova K. K. Hickey K. L. Osuna B. A. Hussmann J. A. Frost A. Weinberg D. E. (2017). CAT-tailing as a fail-safe mechanism for efficient degradation of stalled nascent polypeptides. Science 357 (6349), 414–417. 10.1126/science.aam7787 28751611 PMC5673106

[B79] Kuroha K. Zinoviev A. Hellen C. U. T. Pestova T. V. (2018). Release of ubiquitinated and non-ubiquitinated nascent chains from stalled mammalian ribosomal complexes by ANKZF1 and Ptrh1. Mol. Cell 72 (2), 286–302. 10.1016/j.molcel.2018.08.022 30244831 PMC6344051

[B80] Lakkaraju A. K. Mary C. Scherrer A. Johnson A. E. Strub K. (2008). SRP keeps polypeptides translocation-competent by slowing translation to match limiting ER-targeting sites. Cell 133 (3), 440–451. 10.1016/j.cell.2008.02.049 18455985 PMC2430734

[B81] Lakshminarayan R. Phillips B. P. Binnian I. L. Gomez-Navarro N. Escudero-Urquijo N. Warren A. J. (2020). Pre-emptive quality control of a misfolded membrane protein by ribosome-driven effects. Curr. Biol. 30 (5), 854–864. e855. 10.1016/j.cub.2019.12.060 31956032 PMC7063571

[B82] LaRiviere F. J. Cole S. E. Ferullo D. J. Moore M. J. (2006). A late-acting quality control process for mature eukaryotic rRNAs. Mol. Cell 24 (4), 619–626. 10.1016/j.molcel.2006.10.008 17188037

[B83] Le Hir H. Gatfield D. Izaurralde E. Moore M. J. (2001). The exon-exon junction complex provides a binding platform for factors involved in mRNA export and nonsense-mediated mRNA decay. EMBO J. 20 (17), 4987–4997. 10.1093/emboj/20.17.4987 11532962 PMC125616

[B84] Lelivelt M. J. Culbertson M. R. (1999). Yeast Upf proteins required for RNA surveillance affect global expression of the yeast transcriptome. Mol. Cell Biol. 19 (10), 6710–6719. 10.1128/MCB.19.10.6710 10490610 PMC84660

[B85] Li J. Ding S. C. Cho H. Chung B. C. Gale M. Jr. Chanda S. K. (2013). A short hairpin RNA screen of interferon-stimulated genes identifies a novel negative regulator of the cellular antiviral response. mBio 4 (3), e00385–e00313. 10.1128/mBio.00385-13 23781071 PMC3684836

[B86] Li S. Ikeuchi K. Kato M. Buschauer R. Sugiyama T. Adachi S. (2022). Sensing of individual stalled 80S ribosomes by Fap1 for nonfunctional rRNA turnover. Mol. Cell 82 (18), 3424–3437.e8. 10.1016/j.molcel.2022.08.018 36113412

[B87] Li W. Ward F. R. McClure K. F. Chang S. T. Montabana E. Liras S. (2019). Structural basis for selective stalling of human ribosome nascent chain complexes by a drug-like molecule. Nat. Struct. Mol. Biol. 26 (6), 501–509. 10.1038/s41594-019-0236-8 31160784 PMC6919564

[B88] Lill R. Muhlenhoff U. (2008). Maturation of iron-sulfur proteins in eukaryotes: Mechanisms, connected processes, and diseases. Annu. Rev. Biochem. 77, 669–700. 10.1146/annurev.biochem.76.052705.162653 18366324

[B89] Lin Y. J. Huang L. H. Huang C. T. (2013). Enhancement of heterologous gene expression in Flammulina velutipes using polycistronic vectors containing a viral 2A cleavage sequence. PLoS One 8 (3), e59099. 10.1371/journal.pone.0059099 23516605 PMC3597617

[B90] Lin Y. Li F. Huang L. Polte C. Duan H. Fang J. (2020). eIF3 associates with 80S ribosomes to promote translation elongation, mitochondrial homeostasis, and muscle health. Mol. Cell 79 (4), 575–587. 10.1016/j.molcel.2020.06.003 32589965

[B91] Losson R. Lacroute F. (1979). Interference of nonsense mutations with eukaryotic messenger RNA stability. Proc. Natl. Acad. Sci. U. S. A. 76 (10), 5134–5137. 10.1073/pnas.76.10.5134 388431 PMC413094

[B92] Lyumkis D. Oliveira dos Passos D. Tahara E. B. Webb K. Bennett E. J. Vinterbo S. (2014). Structural basis for translational surveillance by the large ribosomal subunit-associated protein quality control complex. Proc. Natl. Acad. Sci. U. S. A. 111 (45), 15981–15986. 10.1073/pnas.1413882111 25349383 PMC4234556

[B93] Manuvakhova M. Keeling K. Bedwell D. M. (2000). Aminoglycoside antibiotics mediate context-dependent suppression of termination codons in a mammalian translation system. RNA 6 (7), 1044–1055. 10.1017/s1355838200000716 10917599 PMC1369979

[B94] Maquat L. E. Kinniburgh A. J. Rachmilewitz E. A. Ross J. (1981). Unstable beta-globin mRNA in mRNA-deficient beta o thalassemia. Cell 27 (3), 543–553. 10.1016/0092-8674(81)90396-2 6101206

[B95] Martin P. B. Kigoshi-Tansho Y. Sher R. B. Ravenscroft G. Stauffer J. E. Kumar R. (2020). NEMF mutations that impair ribosome-associated quality control are associated with neuromuscular disease. Nat. Commun. 11 (1), 4625. 10.1038/s41467-020-18327-6 32934225 PMC7494853

[B96] Mason N. Ciufo L. F. Brown J. D. (2000). Elongation arrest is a physiologically important function of signal recognition particle. EMBO J. 19 (15), 4164–4174. 10.1093/emboj/19.15.4164 10921896 PMC306590

[B97] Matsuo Y. Ikeuchi K. Saeki Y. Iwasaki S. Schmidt C. Udagawa T. (2017). Ubiquitination of stalled ribosome triggers ribosome-associated quality control. Nat. Commun. 8 (1), 159. 10.1038/s41467-017-00188-1 28757607 PMC5534433

[B98] Matsuo Y. Inada T. (2021). The ribosome collision sensor Hel2 functions as preventive quality control in the secretory pathway. Cell Rep. 34 (12), 108877. 10.1016/j.celrep.2021.108877 33761353

[B99] Meaux S. Van Hoof A. (2006). Yeast transcripts cleaved by an internal ribozyme provide new insight into the role of the cap and poly(A) tail in translation and mRNA decay. RNA 12 (7), 1323–1337. 10.1261/rna.46306 16714281 PMC1484436

[B100] Mendell J. T. Sharifi N. A. Meyers J. L. Martinez-Murillo F. Dietz H. C. (2004). Nonsense surveillance regulates expression of diverse classes of mammalian transcripts and mutes genomic noise. Nat. Genet. 36 (10), 1073–1078. 10.1038/ng1429 15448691

[B101] Mendonsa S. von Kuegelgen N. Bujanic L. Chekulaeva M. (2021). Charcot-Marie-Tooth mutation in glycyl-tRNA synthetase stalls ribosomes in a pre-accommodation state and activates integrated stress response. Nucleic Acids Res. 49 (17), 10007–10017. 10.1093/nar/gkab730 34403468 PMC8464049

[B102] Meydan S. Guydosh N. R. (2020). Disome and trisome profiling reveal genome-wide targets of ribosome quality control. Mol. Cell 79 (4), 588–602. e586. 10.1016/j.molcel.2020.06.010 32615089 PMC7484464

[B103] Miller-Vedam L. E. Brauning B. Popova K. D. Schirle Oakdale N. T. Bonnar J. L. Prabu J. R. (2020). Structural and mechanistic basis of the EMC-dependent biogenesis of distinct transmembrane clients. Elife 9, e62611–e62647. 10.7554/eLife.62611 33236988 PMC7785296

[B104] Mills E. W. Wangen J. Green R. Ingolia N. T. (2016). Dynamic regulation of a ribosome rescue pathway in erythroid cells and platelets. Cell Rep. 17 (1), 1–10. 10.1016/j.celrep.2016.08.088 27681415 PMC5111367

[B105] Muller J. B. Geyer P. E. Colaco A. R. Treit P. V. Strauss M. T. Oroshi M. (2020). The proteome landscape of the kingdoms of life. Nature 582 (7813), 592–596. 10.1038/s41586-020-2402-x 32555458

[B106] Munroe D. Jacobson A. (1990). mRNA poly(A) tail, a 3' enhancer of translational initiation. Mol. Cell Biol. 10 (7), 3441–3455. 10.1128/mcb.10.7.3441 1972543 PMC360780

[B107] Namy O. Hatin I. Rousset J. P. (2001). Impact of the six nucleotides downstream of the stop codon on translation termination. EMBO Rep. 2 (9), 787–793. 10.1093/embo-reports/kve176 11520858 PMC1084031

[B108] Niesen M. J. M. Muller-Lucks A. Hedman R. von Heijne G. Miller T. F. 3rd (2018). Forces on nascent polypeptides during membrane insertion and translocation via the Sec translocon. Biophys. J. 115 (10), 1885–1894. 10.1016/j.bpj.2018.10.002 30366631 PMC6303271

[B109] Nillegoda N. B. Theodoraki M. A. Mandal A. K. Mayo K. J. Ren H. Y. Sultana R. (2010). Ubr1 and Ubr2 function in a quality control pathway for degradation of unfolded cytosolic proteins. Mol. Biol. Cell 21 (13), 2102–2116. 10.1091/mbc.E10-02-0098 20462952 PMC2893976

[B110] Nunomura A. Honda K. Takeda A. Hirai K. Zhu X. Smith M. A. (2006). Oxidative damage to RNA in neurodegenerative diseases. J. Biomed. Biotechnol. 2006 (3), 82323. 10.1155/JBB/2006/82323 17047315 PMC1559934

[B111] O'Donnell J. P. Phillips B. P. Yagita Y. Juszkiewicz S. Wagner A. Malinverni D. (2020). The architecture of EMC reveals a path for membrane protein insertion. Elife 9, 578877–e57930. 10.7554/eLife.57887 PMC7292650 32459176

[B112] Osuna B. A. Howard C. J. Kc S. Frost A. Weinberg D. E. (2017). *In vitro* analysis of RQC activities provides insights into the mechanism and function of CAT tailing. Elife 6, 279499–e28019. 10.7554/eLife.27949 PMC5562442 28718767

[B113] Palmer E. Wilhelm J. M. Sherman F. (1979). Phenotypic suppression of nonsense mutants in yeast by aminoglycoside antibiotics. Nature 277 (5692), 148–150. 10.1038/277148a0 366439

[B114] Partridge A. W. Therien A. G. Deber C. M. (2002). Polar mutations in membrane proteins as a biophysical basis for disease. Biopolymers 66 (5), 350–358. 10.1002/bip.10313 12539263

[B115] Paushkin S. V. Kushnirov V. V. Smirnov V. N. Ter-Avanesyan M. D. (1996). Propagation of the yeast prion-like [psi+] determinant is mediated by oligomerization of the SUP35-encoded polypeptide chain release factor. EMBO J. 15 (12), 3127–3134. 10.1002/j.1460-2075.1996.tb00675.x 8670813 PMC450255

[B116] Pechmann S. Frydman J. (2013). Evolutionary conservation of codon optimality reveals hidden signatures of cotranslational folding. Nat. Struct. Mol. Biol. 20 (2), 237–243. 10.1038/nsmb.2466 23262490 PMC3565066

[B117] Peixeiro I. Inacio A. Barbosa C. Silva A. L. Liebhaber S. A. Romao L. (2012). Interaction of PABPC1 with the translation initiation complex is critical to the NMD resistance of AUG-proximal nonsense mutations. Nucleic Acids Res. 40 (3), 1160–1173. 10.1093/nar/gkr820 21989405 PMC3273812

[B118] Peter J. J. Magnussen H. M. DaRosa P. A. Millrine D. Matthews S. P. Lamoliatte F. (2022). A non-canonical scaffold-type E3 ligase complex mediates protein UFMylation. EMBO J. 41, e111015. n/a(n/a). 10.15252/embj.2022111015 36121123 PMC9627666

[B119] Phillips B. P. Miller E. A. (2020). Ribosome-associated quality control of membrane proteins at the endoplasmic reticulum. J. Cell Sci. 133 (22), jcs251983. 10.1242/jcs.251983 33247003 PMC7116877

[B120] Pisareva V. P. Skabkin M. A. Hellen C. U. Pestova T. V. Pisarev A. V. (2011). Dissociation by Pelota, Hbs1 and ABCE1 of mammalian vacant 80S ribosomes and stalled elongation complexes. EMBO J. 30 (9), 1804–1817. 10.1038/emboj.2011.93 21448132 PMC3101999

[B121] Powers K. T. Szeto J. A. Schaffitzel C. (2020). New insights into no-go, non-stop and nonsense-mediated mRNA decay complexes. Curr. Opin. Struct. Biol. 65, 110–118. 10.1016/j.sbi.2020.06.011 32688260

[B122] Pradhan A. K. Kandasamy G. Chatterjee U. Bharadwaj A. Mathew S. J. Dohmen R. J. (2021). Ribosome-associated quality control mediates degradation of the premature translation termination product Orf1p of ODC antizyme mRNA. FEBS Lett. 595 (15), 2015–2033. 10.1002/1873-3468.14147 34109626

[B123] Rehwinkel J. Letunic I. Raes J. Bork P. Izaurralde E. (2005). Nonsense-mediated mRNA decay factors act in concert to regulate common mRNA targets. RNA 11 (10), 1530–1544. 10.1261/rna.2160905 16199763 PMC1370837

[B124] Robinson K. S. Toh G. A. Rozario P. Chua R. Bauernfried S. Sun Z. (2022). ZAKα-driven ribotoxic stress response activates the human NLRP1 inflammasome. Science 377 (6603), 328–335. 10.1126/science.abl6324 35857590 PMC7614315

[B125] Rodrigo-Brenni M. C. Hegde R. S. (2012). Design principles of protein biosynthesis-coupled quality control. Dev. Cell 23 (5), 896–907. 10.1016/j.devcel.2012.10.012 23153486

[B126] Russell S. J. Steger K. A. Johnston S. A. (1999). Subcellular localization, stoichiometry, and protein levels of 26 S proteasome subunits in yeast. J. Biol. Chem. 274 (31), 21943–21952. 10.1074/jbc.274.31.21943 10419517

[B127] Sato S. Ward C. L. Kopito R. R. (1998). Cotranslational ubiquitination of cystic fibrosis transmembrane conductance regulator *in vitro* . J. Biol. Chem. 273 (13), 7189–7192. 10.1074/jbc.273.13.7189 9516408

[B128] Schubert U. Anton L. C. Gibbs J. Norbury C. C. Yewdell J. W. Bennink J. R. (2000). Rapid degradation of a large fraction of newly synthesized proteins by proteasomes. Nature 404 (6779), 770–774. 10.1038/35008096 10783891

[B129] Sha Z. Brill L. M. Cabrera R. Kleifeld O. Scheliga J. S. Glickman M. H. (2009). The eIF3 interactome reveals the translasome, a supercomplex linking protein synthesis and degradation machineries. Mol. Cell 36 (1), 141–152. 10.1016/j.molcel.2009.09.026 19818717 PMC2789680

[B130] Shao S. Brown A. Santhanam B. Hegde R. S. (2015). Structure and assembly pathway of the ribosome quality control complex. Mol. Cell 57 (3), 433–444. 10.1016/j.molcel.2014.12.015 25578875 PMC4321881

[B131] Shen P. S. Park J. Qin Y. Li X. Parsawar K. Larson M. H. (2015). Protein synthesis. Rqc2p and 60S ribosomal subunits mediate mRNA-independent elongation of nascent chains. Science 347 (6217), 75–78. 10.1126/science.1259724 25554787 PMC4451101

[B132] Shin C. Nam J. W. Farh K. K. Chiang H. R. Shkumatava A. Bartel D. P. (2010). Expanding the microRNA targeting code: Functional sites with centered pairing. Mol. Cell 38 (6), 789–802. 10.1016/j.molcel.2010.06.005 20620952 PMC2942757

[B133] Shoemaker C. J. Eyler D. E. Green R. (2010). Dom34:Hbs1 promotes subunit dissociation and peptidyl-tRNA drop-off to initiate no-go decay. Science 330 (6002), 369–372. 10.1126/science.1192430 20947765 PMC4022135

[B134] Shurtleff M. J. Itzhak D. N. Hussmann J. A. Schirle Oakdale N. T. Costa E. A. Jonikas M. (2018). The ER membrane protein complex interacts cotranslationally to enable biogenesis of multipass membrane proteins. Elife 7, e37018. 10.7554/eLife.37018 29809151 PMC5995541

[B135] Simms C. L. Hudson B. H. Mosior J. W. Rangwala A. S. Zaher H. S. (2014). An active role for the ribosome in determining the fate of oxidized mRNA. Cell Rep. 9 (4), 1256–1264. 10.1016/j.celrep.2014.10.042 25456128 PMC4254665

[B136] Simms C. L. Yan L. L. Zaher H. S. (2017). Ribosome collision is critical for quality control during No-go decay. Mol. Cell 68 (2), 361–373. 10.1016/j.molcel.2017.08.019 28943311 PMC5659757

[B137] Sinha N. K. Ordureau A. Best K. Saba J. A. Zinshteyn B. Sundaramoorthy E. (2020). EDF1 coordinates cellular responses to ribosome collisions. Elife 9, e58828. 10.7554/eLife.58828 32744497 PMC7486125

[B138] Sitron C. S. Brandman O. (2019). CAT tails drive degradation of stalled polypeptides on and off the ribosome. Nat. Struct. Mol. Biol. 26 (6), 450–459. 10.1038/s41594-019-0230-1 31133701 PMC6554034

[B139] Sitron C. S. Brandman O. (2020). Detection and degradation of stalled nascent chains via ribosome-associated quality control. Annu. Rev. Biochem. 89 (1), 417–442. 10.1146/annurev-biochem-013118-110729 32569528 PMC8258965

[B140] Sitron C. S. Park J. H. Brandman O. (2017). Asc1, Hel2, and Slh1 couple translation arrest to nascent chain degradation. RNA 23 (5), 798–810. 10.1261/rna.060897.117 28223409 PMC5393187

[B141] Sitron C. S. Park J. H. Giafaglione J. M. Brandman O. (2020). Aggregation of CAT tails blocks their degradation and causes proteotoxicity in *S. cerevisiae* . PLoS One 15 (1), e0227841. 10.1371/journal.pone.0227841 31945107 PMC6964901

[B142] Smalinskaite L. Kim M. K. Lewis A. J. O. Keenan R. J. Hegde R. S. (2022). Mechanism of an intramembrane chaperone for multipass membrane proteins. Nature 611, 161–166. 10.1038/s41586-022-05336-2 36261528 PMC7614104

[B143] Stein K. C. Kriel A. Frydman J. (2019). Nascent polypeptide domain topology and elongation rate direct the cotranslational hierarchy of Hsp70 and TRiC/CCT. Mol. Cell 75 (6), 1117–1130. 10.1016/j.molcel.2019.06.036 31400849 PMC6953483

[B144] Stein K. C. Morales-Polanco F. van der Lienden J. Rainbolt T. K. Frydman J. (2022). Ageing exacerbates ribosome pausing to disrupt cotranslational proteostasis. Nature 601 (7894), 637–642. 10.1038/s41586-021-04295-4 35046576 PMC8918044

[B145] Stephani M. Picchianti L. Gajic A. Beveridge R. Skarwan E. Sanchez de Medina Hernandez V. (2020). A cross-kingdom conserved ER-phagy receptor maintains endoplasmic reticulum homeostasis during stress. Elife 9, 583966–e59105. 10.7554/eLife.58396 PMC7515635 32851973

[B146] Stoneley M. Harvey R. F. Mulroney T. E. Mordue R. Jukes-Jones R. Cain K. (2022). Unresolved stalled ribosome complexes restrict cell-cycle progression after genotoxic stress. Mol. Cell 82 (8), 1557–1572.e7. e1557. 10.1016/j.molcel.2022.01.019 35180429 PMC9098122

[B147] Sudmant P. H. Lee H. Dominguez D. Heiman M. Burge C. B. (2018). Widespread accumulation of ribosome-associated isolated 3' UTRs in neuronal cell populations of the aging brain. Cell Rep. 25 (9), 2447–2456. 10.1016/j.celrep.2018.10.094 30485811 PMC6354779

[B148] Sugiyama T. Li S. Kato M. Ikeuchi K. Ichimura A. Matsuo Y. (2019). Sequential ubiquitination of ribosomal protein uS3 triggers the degradation of non-functional 18S rRNA. Cell Rep. 26 (12), 3400–3415. e3407. 10.1016/j.celrep.2019.02.067 30893611

[B149] Sundaram A. Yamsek M. Zhong F. Hooda Y. Hegde R. S. Keenan R. J. (2022). Substrate-driven assembly of a translocon for multipass membrane proteins. Nature 611, 167–172. 10.1038/s41586-022-05330-8 36261522 PMC9630114

[B150] Sundaramoorthy E. Leonard M. Mak R. Liao J. Fulzele A. Bennett E. J. (2017). ZNF598 and RACK1 regulate mammalian ribosome-associated quality control function by mediating regulatory 40S ribosomal ubiquitylation. Mol. Cell 65 (4), 751–760. e754. 10.1016/j.molcel.2016.12.026 28132843 PMC5321136

[B151] Sundaramoorthy E. Ryan A. P. Fulzele A. Leonard M. Daugherty M. D. Bennett E. J. (2021). Ribosome quality control activity potentiates vaccinia virus protein synthesis during infection. J. Cell Sci. 134 (8), jcs257188. 10.1242/jcs.257188 33912921 PMC8106952

[B152] Tanaka M. Chock P. B. Stadtman E. R. (2007). Oxidized messenger RNA induces translation errors. Proc. Natl. Acad. Sci. U. S. A. 104 (1), 66–71. 10.1073/pnas.0609737104 17190801 PMC1765478

[B153] Terrey M. Adamson S. I. Gibson A. L. Deng T. Ishimura R. Chuang J. H. (2020). GTPBP1 resolves paused ribosomes to maintain neuronal homeostasis. Elife 9, e62731. 10.7554/eLife.62731 33186095 PMC7665888

[B154] Thrun A. Garzia A. Kigoshi-Tansho Y. Patil P. R. Umbaugh C. S. Dallinger T. (2021). Convergence of mammalian RQC and C-end rule proteolytic pathways via alanine tailing. Mol. Cell 81 (10), 2112–2122.e7. e2117. 10.1016/j.molcel.2021.03.004 33909987 PMC8141035

[B155] Tian G. Hu C. Yun Y. Yang W. Dubiel W. Cheng Y. (2021). Dual roles of HSP70 chaperone HSPA1 in quality control of nascent and newly synthesized proteins. EMBO J. 40 (13), e106183. 10.15252/embj.2020106183 34010456 PMC8246255

[B156] Tian S. Wu Q. Zhou B. Choi M. Y. Ding B. Yang W. (2019). Proteomic analysis identifies membrane proteins dependent on the ER membrane protein complex. Cell Rep. 28 (10), 2517–2526. e2515. 10.1016/j.celrep.2019.08.006 31484065 PMC6749609

[B157] Trentini D. B. Pecoraro M. Tiwary S. Cox J. Mann M. Hipp M. S. (2020). Role for ribosome-associated quality control in sampling proteins for MHC class I-mediated antigen presentation. Proc. Natl. Acad. Sci. U. S. A. 117 (8), 4099–4108. 10.1073/pnas.1914401117 32047030 PMC7049129

[B158] Tsuboi T. Kuroha K. Kudo K. Makino S. Inoue E. Kashima I. (2012). Dom34:hbs1 plays a general role in quality-control systems by dissociation of a stalled ribosome at the 3' end of aberrant mRNA. Mol. Cell 46 (4), 518–529. 10.1016/j.molcel.2012.03.013 22503425

[B159] Turner G. C. Varshavsky A. (2000). Detecting and measuring cotranslational protein degradation *in vivo* . Science 289 (5487), 2117–2120. 10.1126/science.289.5487.2117 11000112

[B160] Udagawa T. Seki M. Okuyama T. Adachi S. Natsume T. Noguchi T. (2021). Failure to degrade CAT-tailed proteins disrupts neuronal morphogenesis and cell survival. Cell Rep. 34 (1), 108599. 10.1016/j.celrep.2020.108599 33406423

[B161] Udy D. B. Bradley R. K. (2022). Nonsense-mediated mRNA decay uses complementary mechanisms to suppress mRNA and protein accumulation. Life Sci. Alliance 5 (3), 2021012177–e202101313. 10.26508/lsa.202101217 PMC8711849 34880103

[B162] Vabulas R. M. Hartl F. U. (2005). Protein synthesis upon acute nutrient restriction relies on proteasome function. Science 310 (5756), 1960–1963. 10.1126/science.1121925 16373576

[B163] van Hoof A. Frischmeyer P. A. Dietz H. C. Parker R. (2002). Exosome-mediated recognition and degradation of mRNAs lacking a termination codon. Science 295 (5563), 2262–2264. 10.1126/science.1067272 11910110

[B164] Verma R. Oania R. S. Kolawa N. J. Deshaies R. J. (2013). Cdc48/p97 promotes degradation of aberrant nascent polypeptides bound to the ribosome. Elife 2, e00308. 10.7554/eLife.00308 23358411 PMC3552423

[B165] Verma R. Reichermeier K. M. Burroughs A. M. Oania R. S. Reitsma J. M. Aravind L. (2018). Vms1 and ANKZF1 peptidyl-tRNA hydrolases release nascent chains from stalled ribosomes. Nature 557 (7705), 446–451. 10.1038/s41586-018-0022-5 29632312 PMC6226276

[B166] von der Haar T. (2008). A quantitative estimation of the global translational activity in logarithmically growing yeast cells. BMC Syst. Biol. 2, 87. 10.1186/1752-0509-2-87 18925958 PMC2590609

[B167] von der Malsburg K. Shao S. Hegde R. S. (2015). The ribosome quality control pathway can access nascent polypeptides stalled at the Sec61 translocon. Mol. Biol. Cell 26 (12), 2168–2180. 10.1091/mbc.E15-01-0040 25877867 PMC4462936

[B168] Walczak C. P. Leto D. E. Zhang L. Riepe C. Muller R. Y. DaRosa P. A. (2019). Ribosomal protein RPL26 is the principal target of UFMylation. Proc. Natl. Acad. Sci. U. S. A. 116 (4), 1299–1308. 10.1073/pnas.1816202116 30626644 PMC6347690

[B169] Walter P. Blobel G. (1981). Translocation of proteins across the endoplasmic reticulum III. Signal recognition protein (SRP) causes signal sequence-dependent and site-specific arrest of chain elongation that is released by microsomal membranes. J. Cell Biol. 91 (2), 557–561. 10.1083/jcb.91.2.557 7309797 PMC2111983

[B170] Wan L. Juszkiewicz S. Blears D. Bajpe P. K. Han Z. Faull P. (2021). Translation stress and collided ribosomes are co-activators of cGAS. Mol. Cell 81 (13), 2808–2822.e10. 10.1016/j.molcel.2021.05.018 34111399 PMC8260207

[B171] Wang F. Durfee L. A. Huibregtse J. M. (2013). A cotranslational ubiquitination pathway for quality control of misfolded proteins. Mol. Cell 50 (3), 368–378. 10.1016/j.molcel.2013.03.009 23583076 PMC3654026

[B172] Wang L. Xu Y. Rogers H. Saidi L. Noguchi C. T. Li H. (2020). UFMylation of RPL26 links translocation-associated quality control to endoplasmic reticulum protein homeostasis. Cell Res. 30 (1), 5–20. 10.1038/s41422-019-0236-6 31595041 PMC6951344

[B173] Wang R. Nambiar R. Zheng D. Tian B. (2018). PolyA_DB 3 catalogs cleavage and polyadenylation sites identified by deep sequencing in multiple genomes. Nucleic Acids Res. 46 (D1), D315–D319. 10.1093/nar/gkx1000 29069441 PMC5753232

[B174] Wangen J. R. Green R. (2020). Stop codon context influences genome-wide stimulation of termination codon readthrough by aminoglycosides. Elife 9, e52611–e52629. 10.7554/eLife.52611 31971508 PMC7089771

[B175] Wiedmann B. Sakai H. Davis T. A. Wiedmann M. (1994). A protein complex required for signal-sequence-specific sorting and translocation. Nature 370 (6489), 434–440. 10.1038/370434a0 8047162

[B176] Williamson L. Saponaro M. Boeing S. East P. Mitter R. Kantidakis T. (2017). UV irradiation induces a non-coding RNA that functionally opposes the protein encoded by the same gene. Cell 168 (5), 843–855. e813. 10.1016/j.cell.2017.01.019 28215706 PMC5332558

[B177] Wilson D. N. Arenz S. Beckmann R. (2016). Translation regulation via nascent polypeptide-mediated ribosome stalling. Curr. Opin. Struct. Biol. 37, 123–133. 10.1016/j.sbi.2016.01.008 26859868

[B178] Winz M. L. Peil L. Turowski T. W. Rappsilber J. Tollervey D. (2019). Molecular interactions between Hel2 and RNA supporting ribosome-associated quality control. Nat. Commun. 10 (1), 563. 10.1038/s41467-019-08382-z 30718516 PMC6362110

[B179] Witting K. F. Mulder M. P. C. (2021). Highly specialized ubiquitin-like modifications: Shedding light into the UFM1 enigma. Biomolecules 11 (2), 255. 10.3390/biom11020255 33578803 PMC7916544

[B180] Wu C. C. Peterson A. Zinshteyn B. Regot S. Green R. (2020). Ribosome collisions trigger general stress responses to regulate cell fate. Cell 182 (2), 404–416. 10.1016/j.cell.2020.06.006 32610081 PMC7384957

[B181] Wu J. Lei G. Mei M. Tang Y. Li H. (2010). A novel C53/LZAP-interacting protein regulates stability of C53/LZAP and DDRGK domain-containing Protein 1 (DDRGK1) and modulates NF-kappaB signaling. J. Biol. Chem. 285 (20), 15126–15136. 10.1074/jbc.M110.110619 20228063 PMC2865345

[B182] Wu Z. Tantray I. Lim J. Chen S. Li Y. Davis Z. (2019). MISTERMINATE mechanistically links mitochondrial dysfunction with proteostasis failure. Mol. Cell 75 (4), 835–848. 10.1016/j.molcel.2019.06.031 31378462 PMC7362879

[B183] Wurtmann E. J. Wolin S. L. (2009). RNA under attack: Cellular handling of RNA damage. Crit. Rev. Biochem. Mol. Biol. 44 (1), 34–49. 10.1080/10409230802594043 19089684 PMC2656420

[B184] Yan L. L. Simms C. L. McLoughlin F. Vierstra R. D. Zaher H. S. (2019). Oxidation and alkylation stresses activate ribosome-quality control. Nat. Commun. 10 (1), 5611. 10.1038/s41467-019-13579-3 31819057 PMC6901537

[B185] Yan L. L. Zaher H. S. (2021). Ribosome quality control antagonizes the activation of the integrated stress response on colliding ribosomes. Mol. Cell 81 (3), 614–628.e4. e614. 10.1016/j.molcel.2020.11.033 33338396 PMC7867595

[B186] Yewdell J. W. Anton L. C. Bennink J. R. (1996). Defective ribosomal products (DRiPs): A major source of antigenic peptides for MHC class I molecules? J. Immunol. 157 (5), 1823–1826. 8757297

[B187] Yonashiro R. Tahara E. B. Bengtson M. H. Khokhrina M. Lorenz H. Chen K. C. (2016). The Rqc2/Tae2 subunit of the ribosome-associated quality control (RQC) complex marks ribosome-stalled nascent polypeptide chains for aggregation. Elife 5, e11794. 10.7554/eLife.11794 26943317 PMC4805532

[B188] Young D. J. Guydosh N. R. Zhang F. Hinnebusch A. G. Green R. (2015). Rli1/ABCE1 recycles terminating ribosomes and controls translation reinitiation in 3'UTRs *in vivo* . Cell 162 (4), 872–884. 10.1016/j.cell.2015.07.041 26276635 PMC4556345

[B189] Yu C. H. Dang Y. Zhou Z. Wu C. Zhao F. Sachs M. S. (2015). Codon usage influences the local rate of translation elongation to regulate Co-translational protein folding. Mol. Cell 59 (5), 744–754. 10.1016/j.molcel.2015.07.018 26321254 PMC4561030

[B190] Zhang G. Ignatova Z. (2011). Folding at the birth of the nascent chain: Coordinating translation with co-translational folding. Curr. Opin. Struct. Biol. 21 (1), 25–31. 10.1016/j.sbi.2010.10.008 21111607

[B191] Zhou M. Fisher E. A. Ginsberg H. N. (1998). Regulated Co-translational ubiquitination of apolipoprotein B100. A new paradigm for proteasomal degradation of a secretory protein. J. Biol. Chem. 273 (38), 24649–24653. 10.1074/jbc.273.38.24649 9733761

[B192] Zhou M. Guo J. Cha J. Chae M. Chen S. Barral J. M. (2013). Non-optimal codon usage affects expression, structure and function of clock protein FRQ. Nature 495 (7439), 111–115. 10.1038/nature11833 23417067 PMC3629845

[B193] Zhu X. Zhang H. Mendell J. T. (2020). Ribosome recycling by ABCE1 links lysosomal function and iron homeostasis to 3' UTR-directed regulation and nonsense-mediated decay. Cell Rep. 32 (2), 107895. 10.1016/j.celrep.2020.107895 32668236 PMC7433747

[B194] Zuko A. Mallik M. Thompson R. Spaulding E. L. Wienand A. R. Been M. (2021). tRNA overexpression rescues peripheral neuropathy caused by mutations in tRNA synthetase. Science 373 (6559), 1161–1166. 10.1126/science.abb3356 34516840 PMC8856733

[B195] Zurita Rendon O. Fredrickson E. K. Howard C. J. Van Vranken J. Fogarty S. Tolley N. D. (2018). Vms1p is a release factor for the ribosome-associated quality control complex. Nat. Commun. 9 (1), 2197. 10.1038/s41467-018-04564-3 29875445 PMC5989216

